# Speciation without Pre-Defined Fitness Functions

**DOI:** 10.1371/journal.pone.0137838

**Published:** 2015-09-15

**Authors:** Robin Gras, Abbas Golestani, Andrew P. Hendry, Melania E. Cristescu

**Affiliations:** 1 School of Computer Science, University of Windsor, Windsor, ON, Canada; 2 Department of Biology, University of Windsor, Windsor, ON, Canada; 3 Great Lakes Institute for Environmental Research, Windsor, ON, Canada; 4 Redpath Museum & Department of Biology, McGill University, Montreal, QC, Canada; University of Arkansas, UNITED STATES

## Abstract

The forces promoting and constraining speciation are often studied in theoretical models because the process is hard to observe, replicate, and manipulate in real organisms. Most models analyzed to date include pre-defined functions influencing fitness, leaving open the question of how speciation might proceed without these built-in determinants. To consider the process of speciation without pre-defined functions, we employ the individual-based ecosystem simulation platform EcoSim. The environment is initially uniform across space, and an evolving behavioural model then determines how prey consume resources and how predators consume prey. Simulations including natural selection (i.e., an evolving behavioural model that influences survival and reproduction) frequently led to strong and distinct phenotypic/genotypic clusters between which hybridization was low. This speciation was the result of divergence between spatially-localized clusters in the behavioural model, an emergent property of evolving ecological interactions. By contrast, simulations without natural selection (i.e., behavioural model turned off) but with spatial isolation (i.e., limited dispersal) produced weaker and overlapping clusters. Simulations without natural selection or spatial isolation (i.e., behaviour model turned off and high dispersal) did not generate clusters. These results confirm the role of natural selection in speciation by showing its importance even in the absence of pre-defined fitness functions.

## Introduction

Darwin’s ‘mystery of mysteries,’ the origin of species, is difficult to study in nature because–in most cases–the process is relatively rare, protracted, and unreplicated [1]. Mechanisms of speciation–and the forces influencing them–are therefore often studied in theoretical models [2–5]. These models can be grouped into several broad classes–a summary of which will set the stage for illustrating how our model differs. {1} A single starting population is subjected to a pre-defined intra-specific competition function on a pre-defined resource distribution that would favour a single phenotype in the absence of competition: i.e., ‘adaptive or competitive speciation’ [6, 7]. {2} Spatially isolated populations, with or without gene flow, are subject to different selective environments, which are typically specified *a priori* as favouring or disfavouring particular phenotypes or genotypes: i.e., ‘ecological speciation’ [7–9]. {3} Spatially isolated populations are subject to a single pre-defined selective pressure (or no selection at all), in response to which they can evolve different and incompatible mutations: i.e., ‘mutation order speciation’ [10]. {4} Different groups are subject to similar pre-defined natural selection but different patterns of sexual selection, which can be pre-defined or can evolve owing to pre-defined fitness consequences [11, 12].

Previous speciation models thus take a diversity of forms and are implemented in a diversity of ways; yet a feature common to most of them, as we have emphasized above, is reliance at some stage on pre-defined fitness functions or strict constraints on the size of the model. This reliance on investigator-specified functions and constraints leaves open the possibility that the outcomes are dependent on these constraints (see further discussion regarding pre-defined functions and constraints in the **S1 Materials**). Thus, although existing models have taught us much about speciation, they have left open the question of how speciation proceeds in the absence of pre-defined functions. To address this key knowledge gap, we here use individual-based simulations to explore speciation in the absence of pre-defined functions. In our model, speciation must instead proceed owing to emergent properties of interactions between individuals in spatial landscapes where abiotic parameters are initially invariant.

## Material and Methods

With increasing computational power, individual-based simulation platforms such as Tierra, Avida, Polyworld, and EcoSim [13–16] can be used to address difficult questions in biology [17–20]. EcoSim [16], in particular, has been designed to model large-scale virtual ecosystems.

We here explain EcoSim using the updated 7-points Overview, Design concepts, and Details (ODD) standard protocol [21] for describing individual-based models. Note that most of the materials in this section have been published in [16].

### Purpose

EcoSim is an individual-based predator-prey simulation designed to simulate individuals’ behavior in a dynamic, evolving ecosystem [16]. The main purpose of EcoSim is to study biological and ecological theories by constructing a complex adaptive system that leads to a generic virtual ecosystem with behaviors similar to those found in nature. It incorporates three trophic levels: primary producers (grass), primary consumers (prey), and top predators. EcoSim uses a fuzzy cognitive map (FCM) to model each individual behavior. Since the FCM is coded in the genome, behavior can evolve during the simulation. Importantly, the fitness of a given set of behaviours is not set. Instead, fitness emerges from interactions between the model organisms and their biotic environment. As just one example, a prey behavioural model could have high fitness if it gives preference to foraging over reproduction when food is sparse (energy reserves therefore low) but gives preference to reproduction over foraging when food is abundant (energy reserves therefore high).

### Entities, State Variables, and Scales

The model has two types of individuals: predators and prey. Each individual possesses a set of life-history characteristics, such as age, minimum age for breeding, speed, vision distance, level of energy, and amount of energy transmitted to the offspring. Energy is provided to individuals by the resources (food) found in their environment. Prey consume primary resources, which are dynamic in quantity and location, whereas predators hunt for prey or scavenge for dead prey (in the following called ‘carrion’). Each individual performs one unique action during a given time step, based on its perception of the environment. Each individual possesses its own FCM coded in its genome, and its behaviors are determined by the interaction between the FCM and the environment. FCMs are weighted graphs representing the causal relationship between stimulus, drive, and activity nodes. Prey individuals gain 250 units of energy by eating one unit of grass, and predators gain 500 units of energy by eating one prey or one unit of carrion. At each time step, an individual spends energy depending on its action (e.g., breeding, eating, running) and on the complexity of its behavioral model (number of existing edges in its FCM). On average, a movement action, such as escape or exploration, requires 50 units of energy, whereas a reproduction action requires 110 units of energy and no action at all results in a small expenditure of 18 units.

The smallest units of the environment are cells. Each cell contains some amount of food and can host an unlimited number of individuals (of course, the actual number will be limited by food). The virtual world consists of a 1000 × 1000 matrix of cells that wraps around in a torus to remove any spatial bias.

Each time step involves each individual perceiving its environment, making a decision, and performing one action; in addition species memberships, including speciation events, are updated and all relevant variables are recorded (e.g., quantity of available food). One generation corresponds to the number of time steps for an individual to reach the age of reproduction (6 for prey and 8 for predators). In terms of computational time, the speed of a simulation per time step is proportional to the number of individuals. On average, at each time step, about 250,000 individuals exist in the world as members of one or several species. A species is a set of individuals with a similar genome relative to a threshold, as will be described below in more detail.

### Process Overview and Scheduling

All the individuals first perceive their environment (all the surrounding cells in their vision range) before using their behavioral model to choose a single action. The possible actions for the prey individuals are: evasion (escape from predator), search for food (if not enough grass is available in the current cell, prey can move to a nearby cell to search for grass), socialization (moving to the closest prey in the vicinity), exploration (random movement), resting (to save energy), eating, and breeding. Predators similarly choose an action from amongst: searching for food, hunting (catching and eating prey), scavenging (eating dead prey = ‘carrion’), socialization, exploration, resting, and breeding. After each action by predators or prey, an individual’s energy is adjusted and its age is incremented by one unit. If the energy level of an individual is less than or equal to zero, the individual dies. After all individuals sequentially perform their actions, the amount of grass and carrion (dead prey) in each cell is adjusted, and the value of the state variables of individuals and cells are updated (section 2–1 in **S1 Materials**).

### Design Concepts

#### Basic principles

To observe the evolution of individual behaviour and, ultimately, the entire ecosystem over thousands of generations without pre-defined fitness functions, the following features were implemented in the model: {1} each individual possesses genomic information; {2} this information influences individual behaviour and, consequently, fitness; {3} the inheritance of genetic material allows for modification (i.e., mutation); {4}the number of individuals is sufficiently high to allow for complex interactions and spatial configurations to emerge; {5} Species are identified based on a measure of genomic similarity; and {6} the number of time steps is. These complex conditions pose computational challenges that require the use of models that combine compactness and ease of computation with a high potential for complex representation.

**Fig 1:**
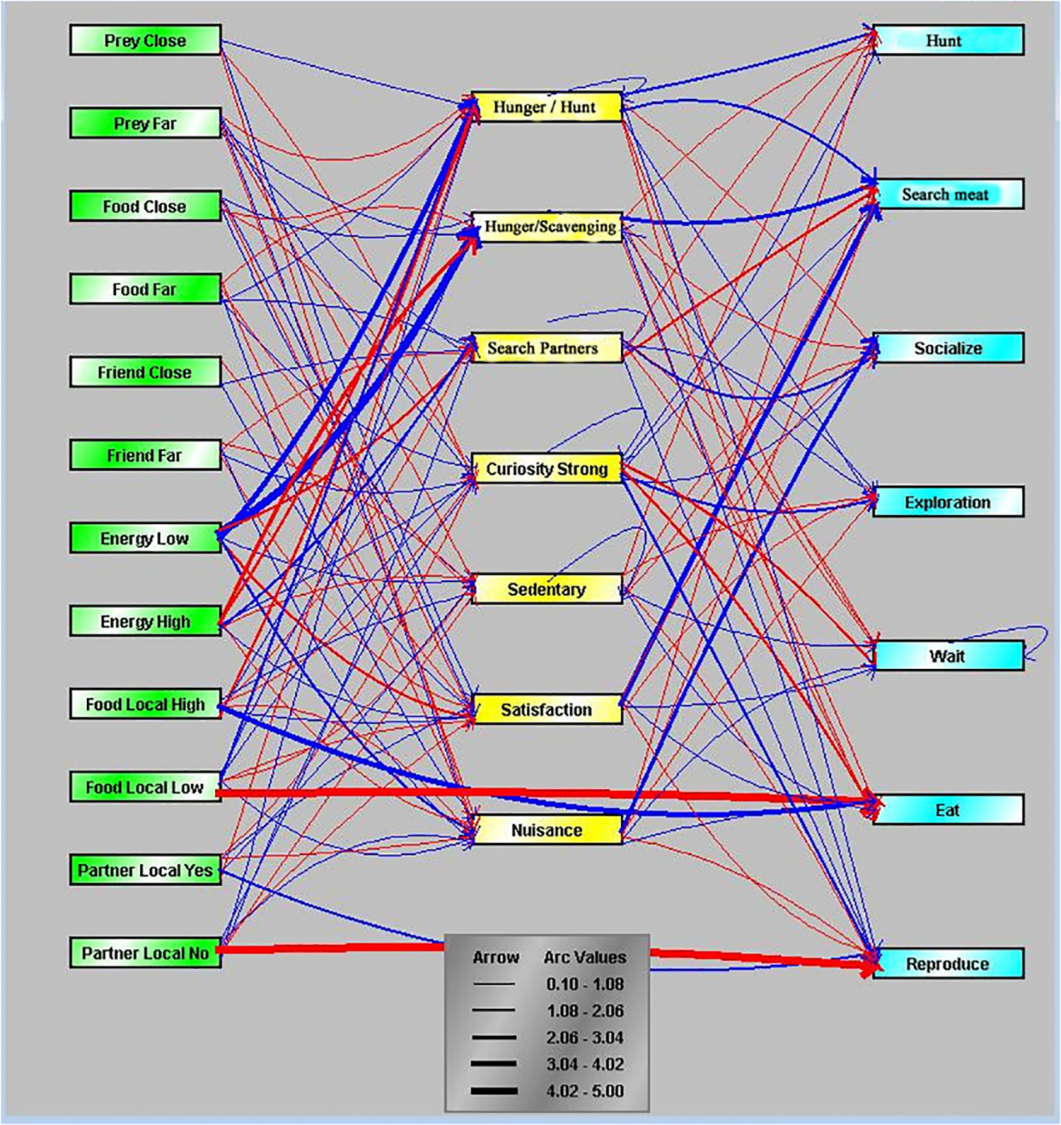
A sample of a predator’s FCM including nodes (left: stimuli, middle: drives, right: activities) and edges. The width of each edge shows the influence value of that edge. Color of edges shows inhibitory (red) or excitatory (blue) effects.

In EcoSim, a Fuzzy Cognitive Map (FCM) [22] is the base for describing and computing individual behaviours. Each individual possesses an FCM (Fig 1) to compute its next action. The FCM is integrally coded in the genome and, therefore, is heritable, mutable and subject to evolution. When a new offspring is created, it receives a genome that combines the genomes of its parents with some possible mutations.

Formally, an FCM is a graph that contains a set of nodes C and a set of edges I, with each edge I_ij_ representing the influence of node C_i_ on node C_j_. A positive weight associated with the edge I_ij_ corresponds to excitation of node C_j_ by node C_i_, whereas a negative weight corresponds to inhibition. If I_ij_ = 0, there is no edge between C_i_ and C_j_ (no influence of Ci on Cj).

#### Emergence

**Fig 2:**
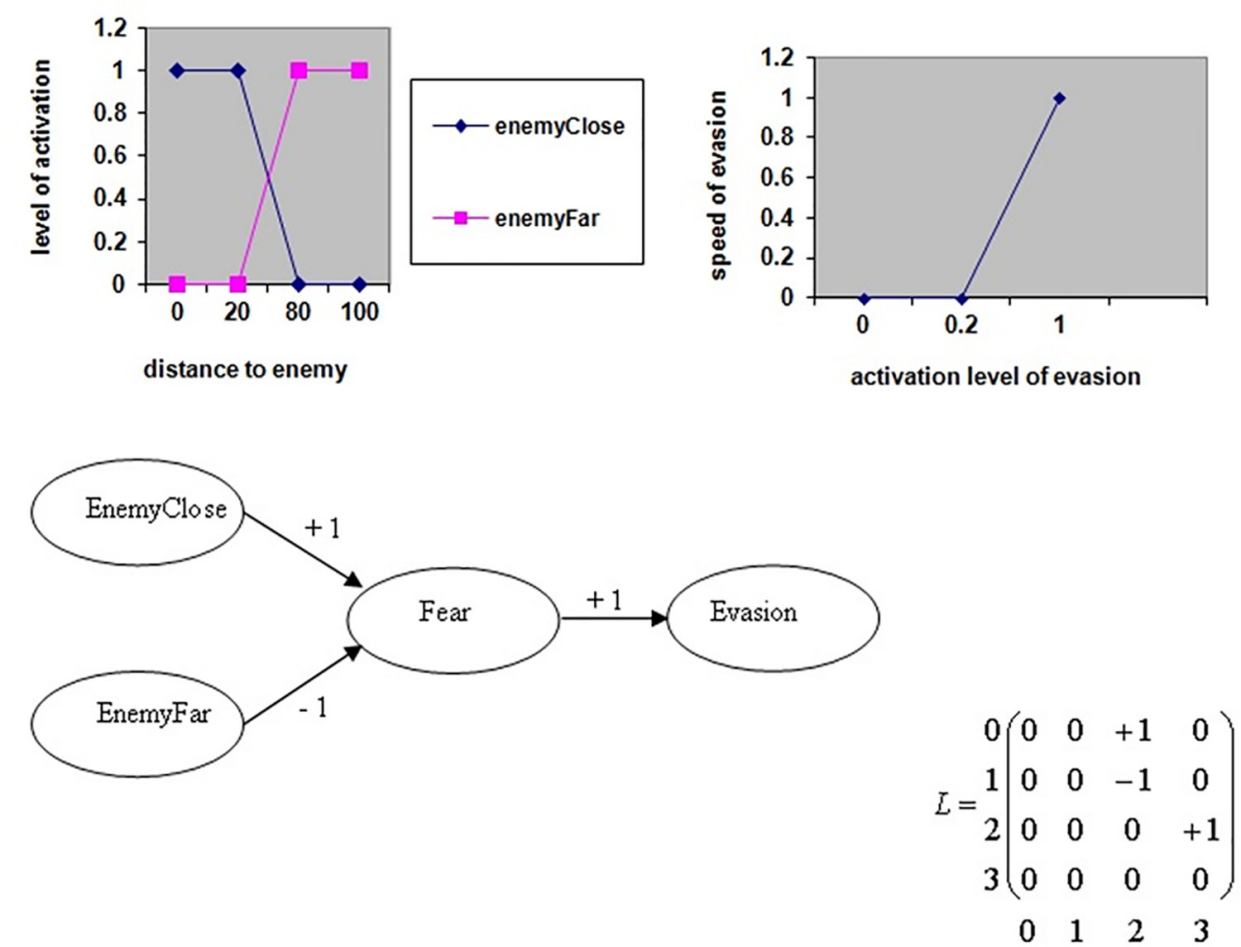
An example of simple FCM for the detection of enemies (predators) and the decision to evade, with its corresponding matrix (0 for ‘Enemy close’, 1 for ‘Enemy far’, 2 for ‘Fear’ and 3 for ‘Evasion’) and the fuzzification (top left) and defuzzification (top right) functions [31].

In each FCM, three kinds of nodes are defined: stimuli (such as distance to ennemy or food, amount of energy, etc.), drives (fear, hunger, curiosity, satisfaction, etc.), and activities (evasion, socialization, exploration, breeding, etc.). The activation level of a stimulus node is computed by performing a fuzzification of the information the individual perceives in the environment (changing its real scalar value into a fuzzy value, i.e., transforming the input value by a potentially non-linear function). For a drive or activity node, C, the activation level is computed from the weighted sum of the current activation level of all input nodes by applying a defuzzification function (another non-linear function transforming the fuzzy input value into the final 'real' value). These fuzzification/defuzzification mechanisms allow for non-linear transformations of the perception signal, which permit, for example, to represent a saturation of information. Finally, the action of an individual is selected based on activity node with the highest activation level. For example, Fig 2 represents two stimulus nodes (enemyClose and enemyFar), one drive (fear), and one activity (evasion). Three influence edges are present: closeness to an enemy excites fear, distance to a enemy inhibits fear, and fear causes evasion. Activations of the nodes enemyClose and enemyFar are computed by fuzzification of the real value of the distance to the enemy, and the defuzzification of the activation of evasion tells us about the speed of the evasion.

**Fig 3:**
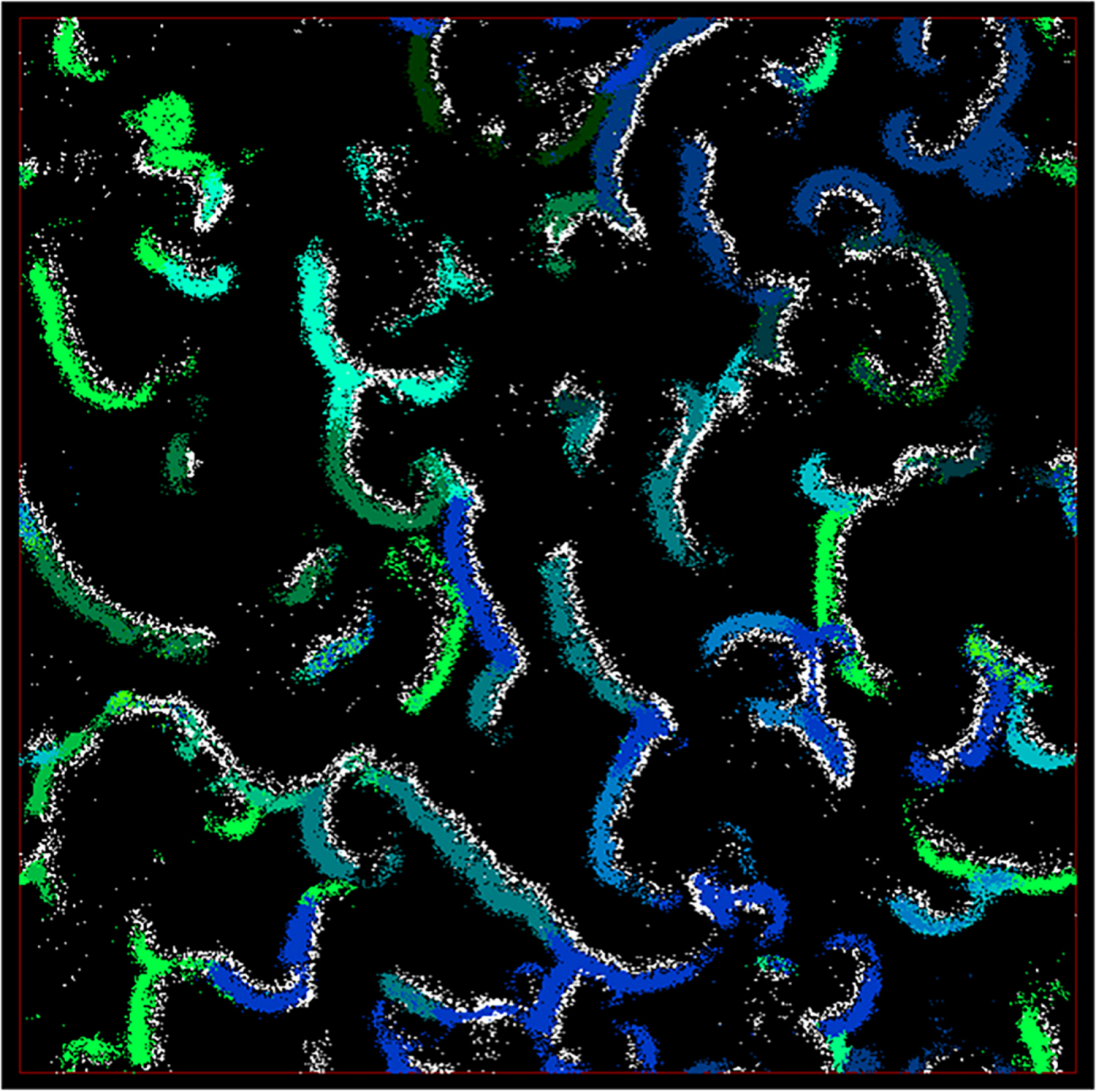
A snapshot of the virtual world in time step 5000. White dots represent predator individuals and the other colors show different prey species.

At initiation of the simulation, prey and predators are scattered randomly across the virtual world (see Table 1). As the simulation proceeds, the distribution of individuals changes based on many factors: prey escaping from predators, individuals socializing and forming groups, individuals migrating to find sources of food, species emerging, etc. The size of the world is large enough to accommodate various population structures and the emergence of migration (i.e. long term global movements of populations across the world). For example, an individual moving at its maximum speed could cross less than half of one dimension of the world during its life span. Moreover, previous studies demonstrate that the use of behavioral models leads to a non-random distribution of individuals into populations/species that contain individuals with similar genomes [20]. Fig 3 shows an example of a snapshot of the virtual world after thousands of time steps with emerging populations.

**Table 1:** Values for user-specified parameters.

User specified parameters	Used value
Initial Number of Prey	12000
Initial Number of Predators	500
Initial Grass Quantity	5790000
Maximum Age Prey	46
Maximum Age Predator	42
Prey Maximum Speed	6
Predator Maximum Speed	11
Prey maximum Energy	650
Predator maximum Energy	1000
Distance for Prey Vision	20
Distance for Predator Vision	25
Reproduction Age for Prey	6
Reproduction Age for Predator	8

It has been shown that the data generated by EcoSim present the same kind of multifractal properties as those observed in real ecosystems [23, 24]: with one example being spiral waves of predator-prey interactions. In fact, strong and robust spiral waves are a common phenomena among complex and dynamic biological systems [25]. Self-organized spiral patterns have been seen not only within chemical reactions but also among populations of bacteria [25] and snowshoe hares in Northern Canada [26]. Prey near the wave break have the capacity to escape from the predators sideways. A subpopulation of prey then finds itself in a region relatively free from predators. In this predator-free zone, prey populations expand extensively, forming a circularly expanding region. The same spiral formation will arise in this new subpopulation of prey and predators, leading to the formation of a second scale [27]. This process repeats many times and the result of this repetition is the emergency of self-similarity [28] in the spatial distribution of individuals.

#### Adaptation

Individuals carry a haploid genome of maximum length if 390 sites, where each site (gene) corresponds to an edge between two nodes of the FCM. However, to allow evolution, many edges have an initial value of zero, and only 114 edges for prey and 107 edges for predators are set at initialization. An additional site is used to code for the amount of energy transmitted from the parent to its offspring at birth. Each gene follows the continuum-of-alleles model and can take values between -12 and +12. These alleles represent the strength of the positive or negative influence of one node on another, such as the strength of the association between a level of hunger and the tendency to feed. The genome of an individual is transmitted to its offspring after being combined with the genome of the other parent and following possible mutations. EcoSim incorporates genetic recombination through crossover and includes epistasis (e.g., multiple stimuli can influence a given drive) but no pleiotropy (each gene influences only one link between nodes). To model simple linkage, alleles are transmitted by blocks: for each node, the values of all its incident edges (in edges) are transmitted together from the same randomly chosen parent (i.e., no recombination among genes for edges to a given node). The probability of mutation is 0.005 per gene and per time step, and the effect of a given mutation is drawn from a normal distribution N(0, 0.1). In addition, a new gene (a new link between nodes) can arise or be lost at a per-generation per-gene probability of 0.001. In this way, new genes can emerge from among the 265 initial edges of zero value.

#### Fitness

To measure the capacity of an individual to survive and produce offspring that can also survive, fitness was calculated as the sum of the ages at death of the individual and its children. It has been shown that this fitness is equivalent as the fitness calculated as the sum of the number of offspring and of offspring of offspring [29]. This was a post-processing computation that was not considered during the simulation.

#### Prediction

The only information available for an individual to make decisions is coming from its perceptions at a particular time step and the values of the activation levels of the drive and activity nodes at the previous time step. Since activation levels are never reset during an individual’s life, its current state depends on all previous states, meaning that the individual has a basic memory of its own past that will influence its future behavior.

#### Sensing

**Fig 4:**
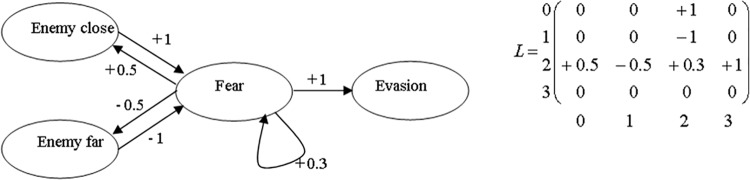
An FCM for the detection of enemies (predators) with its corresponding matrix (0 for ‘Enemy close’, 1 for ‘Enemy far’, 2 for ‘Fear’ and 3 for ‘Evasion’), illustrating the difference between perception and sensation [15].

Each individual in EcoSim is able to sense its local environment inside its range of vision. For instance, each prey can sense its five closest enemies (predators), its five closest cells with food units and its five closest mates within its range of vision, as well as the number of grass units and the potential mates in its current cell. Each individual is also capable of recognizing its current level of energy. Note that the FCM process explained in section **Emergence** distinguishes between perception and sensation: sensation is the real value coming from the environment, whereas perception is sensation modified by an individual’s internal state. For example, it is possible to add three edges to the map in Fig 2: one auto-excitatory edge from the node fear to itself, one excitatory edge from fear to enemyClose, and one inhibitory edge from fear to enemyFar (Fig 4). A given real distance to the enemy thus seems higher or lower depending on the activation level of fear. Also, the fact that the individual is frightened at time t influences the level of fear at time t + 1, which allows modeling the degree of stress. It also enables the individual to memorize information from previous time steps: fear maintains fear. Thus, an FCM can accommodate very complex dynamical systems involving feedback and memory, which is necessary to model complex and evolving behaviors.

#### Interaction

The only interaction that requires a coordinated decision by two individuals is reproduction. For reproduction to be successful, the two parents need to be in the same cell, have sufficient energy and choose the reproduction action. In one of our experiments, there is also ‘enforced reproductive isolation’, where reproduction fails if the two parents are genetically too dissimilar (no extra energy is lost when reproduction fails.) Another interaction is predation. A predator hunting action always succeeds as soon as it can reach the cell of its prey. We assume that a prey generates two units of carrion, one of which is consumed by the predator. Therefore, a new carrion unit is added to the cell, and the energy level of the predator is also increased by one unit of carrion energy. A final interaction is competition for food. For example, if a given cell contains only one food unit and two individuals have chosen the action of eating, it is the younger one that will eat and the older one will not. This is a way to simulate senescence, where older individuals have decreased performance relative to younger individuals. However, relaxing this constraint does not affect our results.

#### Stochasticity

To produce variability in the ecosystem simulation, several processes involve stochasticity. For instance, at initialization, the number of grass units is determined for each cell following an uniform random distribution. Moreover, the maximum age of an individual is determined randomly at birth from a uniform distribution centered at a value which depends on the individual’s type (see Table 1 and Table D in S1 Materials). Stochasticity is also included in several kinds of actions of individuals, such as evasion and socialization. For instance, if no predator or partner is in the vision range, the direction of movement will be random. Furthermore, the direction of the exploration action is always random. However, to understand the extent of randomness in EcoSim, Golestani et al. (2010) examined whether chaotic behavior (one signal of non-randomness) exists in time series generated by the simulation [30]. The authors concluded that the overall behavior of the simulation generates patterns that are non-random and instead represent simple complex biological systems [31].

#### Collectives

EcoSim implements a species concept directly related to the genotypic cluster definition [32], in which a species is a set of individuals sharing a high level of genomic similarity. Each species is then associated with the average of the genetic characteristics of its members, called the ‘species genome’ or the ‘species center’. Over time, a species will progressively contain individuals that are increasingly genetically dissimilar up to an arbitrary threshold where the species splits. This speciation event is inferred from a 2-means clustering algorithm [33] (section 3–1 in **S1 Materials**) determining clusters of individuals that are mutually most similar. After splitting, the two sister species remain sufficiently similar that hybridization can occur until their genomic distance becomes at least half of the speciation threshold (in the model with enforced reproductive isolation). The information about species membership is only a label. It is not used for any purpose during the simulation (e.g. there is no species recognition) but only for post-processing analysis of the results.

#### Observation

EcoSim produces a large amount of data at each time step, including the number of individuals, the characteristics of each individual, and the status of each cell of the virtual world. Information regarding individual characteristics includes spatial position, level of energy, choice of action, species identity, parents, FCM, etc.

### Initialization and Input Data

At initialization, the grass was randomly uniformly distributed (i.e., no divergent selection was imposed across space) and all individuals were genetically identical (with a user defined genome). Other parameter values used in this paper are presented in Table 1.

### Randomized Version of EcoSim

To evaluate the effect of natural selection, we needed a control simulation where natural selection did not occur. To implement this control, we used a random-walk model with no intelligent behaviour of individuals [34]. That is, the behavioural was switched off and possible actions were limited to movement and reproduction. For example, the movement of individuals was random; however, the distribution of movement distances and the size of the world were kept the same as in the other ‘non-control’ simulations. The predator-prey dynamics were determined by Lotka-Volterra competition [35–37]:

$$\begin{array}{l} \frac{dn_{1}}{dt}=r_{1}.\left(1-\frac{n_{1}}{k_{1}}\right).n_{1}-a_{1}.n_{1}.n_{2}\\ \frac{dn_{2}}{dt}=r_{2}.n_{2}+a_{2}.n_{1}.n_{2} \end{array}$$

where n_1_ is the number of prey, n_2_ is the number of predators, dn_1_/dt and dn_2_/dt represent population growth (or decline), t represents time, and r_1_, a_1_, r_2_, a_2_ and k_1_ are parameters representing the interaction between predators and prey set to 0.25, 0.0125, 0.034, 0.21 and 210,000 respectively.

The individuals to die were selected randomly. Moreover, reproduction was also random and, thus, ignored the genetic-similarity requirement specified above (no enforced reproductive isolation). In addition, the locations of the parents and of the offspring were randomly chosen in the high dispersal version. In contrast, in the low dispersal version, the offspring were assigned to the location of one of their two parents. For the sake of consistency, all initial parameters were identical, or as close as possible, to those in the non-randomized runs, and parameters for the Lotka-Volterra model were chosen to induce the same dynamics (average numbers of individuals over time).

This version of EcoSim is similar to the model use in [38]. In their model, an initial population of genetically identical haploid individuals is uniformly distributed in a 2D lattice. Then, the individuals die or reproduce with a fix probability at every time step. For reproduction, a seeker individual randomly selects a mate which has a genetic similarity with itself greater then a fix threshold, likewise implementing an “enforced reproductive isolation” mechanism. The resulting offspring receives a genome which is a combination of the genomes of the two parents plus possible mutations. Their model does not allow the individual to move and therefore force the relation between genetic composition and spatial distribution. Gravrilets [39] proposed a similar model with an “enforced reproductive isolation” mechanism. However, given that his model represents populations and not individuals, it cannot be used to evaluate if the emergence of large sets of mutually isolated populations of genomes is possible because these populations are forced by the model. Therefore, even though these two models have no pre-defined fitness function, they rely on to many constraints and simplifications to be suitable for studying the emergence of species.

### Experimental Design

To investigate the forces influencing speciation, we considered the formation of genetic clusters and the level of hybridization among them. Four main forces could lead to clusters with limited hybridization: {1} enforced reproductive isolation due to a rule that allows only genetically similar individuals to mate, {2} spatial isolation due to low dispersal ability, {3} natural selection as a result of behavioural divergence that causes hybrids to have low fitness (inappropriate combinations of behaviours), and {4} genetic drift where the persistence of the new mutations is governed by chance and these mutations become clustered owing to dispersal limitation. To analyze these potential contributors to speciation, we conducted five experiments in EcoSim.

The first experiment (**Selection, Enforced Reproductive Isolation, and Low Dispersal**) maintained the four forces implemented in Gras et al. (2009) [16] as defined above (see Table 2), including ‘enforced reproductive isolation’ according to genetic similarity (the mating-by-genetic-similarity rule defined in section **Interaction**). Enforced reproductive isolation was absent from all other experiments, which thus lacked this (and any other) pre-defined fitness function.

**Table 2:** Overview of the five experiments and their respective features.

Experiment	Enforced reproductive isolation	Spatial isolation	Natural selection
1. Selection, Enforced Reproductive Isolation, and Low Dispersal	Yes	Yes	Yes
2. Selection and Low Dispersal	No	Yes	Yes
3. Selection and High Dispersal	No	No	Yes
4. No Selection and High Dispersal	No	No	No
5. No Selection and Low Dispersal	No	Yes	No

In the second experiment (**Selection and Low Dispersal**), enforced reproductive isolation was absent, but selection (that leads to evolving FCMs) and low dispersal were retained. The relatively low dispersal ability of individuals allowed for strong spatial clustering and can potentially enhance FCM divergence and, thus, speciation (Table 2).

In the third experiment (**Selection and High Dispersal**), enforced reproductive isolation was absent, but selection was present, and high dispersal across the virtual world facilitated high levels of gene flow. In this simulation, newborn individuals were placed in randomly chosen cells instead of in the cell of its parents. Comparison of the experiments with low and high dispersal allows an analysis of the effects of selection on speciation with and without the potentially enhancing effects of spatial structuring (geographic isolation).

In the fourth experiment (**No Selection and High Dispersal)**, we implemented the randomized version of EcoSim (see section Randomized version of EcoSim) that turns off the behavioural model and replaces it with random activities. Thus, enforced reproductive isolation, spatial isolation, and selection are all deactivated, and the other parameters are kept as close as possible to those of the first experiment. Evolution in this experiment will be driven only by mutation and genetic drift.

In the final experiment (**No Selection and Low Dispersal**) we retained random selection as in experiment 2 but forced the creation of groups of individuals as compact as those in the first and second experiment. To enforce this grouping, we placed the new-born individuals in one of the parent's cells. Otherwise, movement of individuals was random, because individuals did not use their behavioral model.

We conducted 10 simulations for each of the above five experiments. Whereas the first two experiments involve a complex and evolvable behavioral model that allows individuals to make decisions influencing their survival and reproductive success, the last two experiments have individuals making random decisions. For simplicity, we present results only for prey; however, similar results (not shown) are seen for predators.

In total, we conducted 50 independent runs, 10 for each experiment, with an overall computational time of 65,000 hours and about 175 TB (Terabytes) of data. Owing to the complexity of the model and the required computation time (2 processor-months per simulation), we could not analyze multiple parameter combinations in detail. We, therefore, started by exploring various parameter combinations in limited runs to establish sets of values that yielded stable outcomes (i.e., runs with no extinction of all prey or predators during the first 1000 time steps). Similar outcomes were obtained for all of the parameter combinations that yielded stable runs, increasing confidence in the generality of our findings. In particular, this parameter exploration showed that the speciation distance mostly affects the speed at which the observed pattern establishes, not the pattern itself. In this way, we selected a single representative parameter combination (**S1 Materials**).

## Results and Discussion

To explore the causality of species formation, we first investigated the conditions that led to the emergence of strong genetic clusters. EcoSim tests for such clusters, called species-clusters, by implementing a heuristic divisive hierarchical clustering process for all individuals in the entire virtual world at a given time step (section 2–3 in **S1 Materials**). We then evaluated the emergent clusters based on their compactness and separation from other clusters, and also compared these results to those obtained through a K-means-clustering algorithm and through randomized clusters. A good way to assess the organization of the emerging genotype groups is the number of individuals per cluster: if genotype groups exist, then the simulations should generate and maintain clusters with many individuals. Other measures of compactness and separation such as genomic distance between and within clusters and the Davies-Bouldin index (a combination of the two previous measures) are detailed in the **S1 Materials**. All these comparisons and associated statistical tests were performed on the average and standard deviations of ten runs sampled at time steps 12000, 14000, 16000, 18000, and 20000. Our key results are: {1} all experiments involving natural selection (i.e., an evolving behavioural model) led to compact and distinct clusters, {2} experiment with selection but without spatial isolation generated clusters less compact and more overlapping than in the experiments with spatial isolation but the differences were not statistically significant; and {3} experiments with genetic drift alone did not generate clusters. In the following paragraphs, we explore these outcomes–and their implications–in more detail.

**Fig 5:**
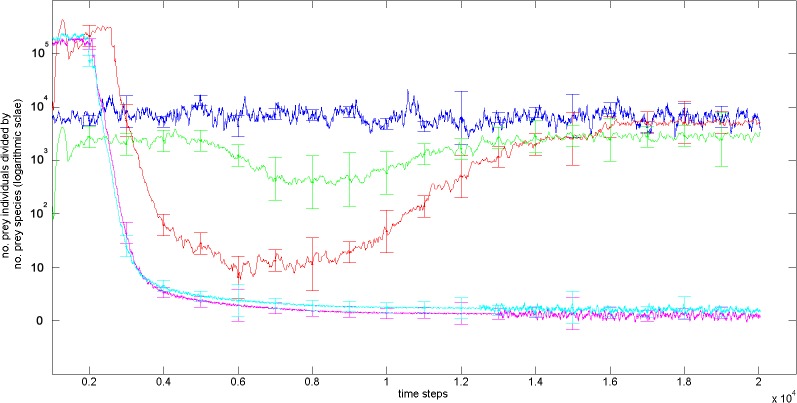
The number of individuals per species (logarithmic scale) in the different simulation experiments (blue line, Selection, Enforced Reproductive Isolation and Low Dispersal experiment; red line, Selection and Low Dispersal experiment; green line, Selection and High Dispersal experiment; clay line, Selection and Low Dispersal experiment; magenta line, No Selection and High Dispersal experiment). The higher stability of **Selection in Enforced Reproductive Isolation and Low Dispersal** compared to the four other experiments is due to the enforced reproductive isolation.

In the experiments with natural selection, the number of individuals per species was much higher than in the experiments without natural selection from time step 10,000 (one-way ANOVA for all considered time steps, *P* = 0.0001; Tukey post hoc test, *P* < 0.05; Fig 5). Moreover, the results for the **Selection and High Dispersal** and the **Selection and Low Dispersal** experiments eventually (from time step 14000) converged toward those obtained for the **Selection, Enforced Reproductive Isolation and Low Dispersal** experiment (around 55 species with several thousand individuals per species, see Table 3). This convergence indicates that the three experiments involving natural selection exhibit the same long-term patterns.

**Table 3:** Average and standard deviation of the number of species for every experiment.

Experiment	Number of Species (Mean)	Number of Species (std)
1. Selection, Enforced Reproductive Isolation, and Low Dispersal	44	8
2. Selection and Low Dispersal	54	11
3. Selection and High Dispersal	62	10
4. No Selection and High Dispersal	65600	75
5. No Selection and Low Dispersal	66100	87

**Fig 6:**
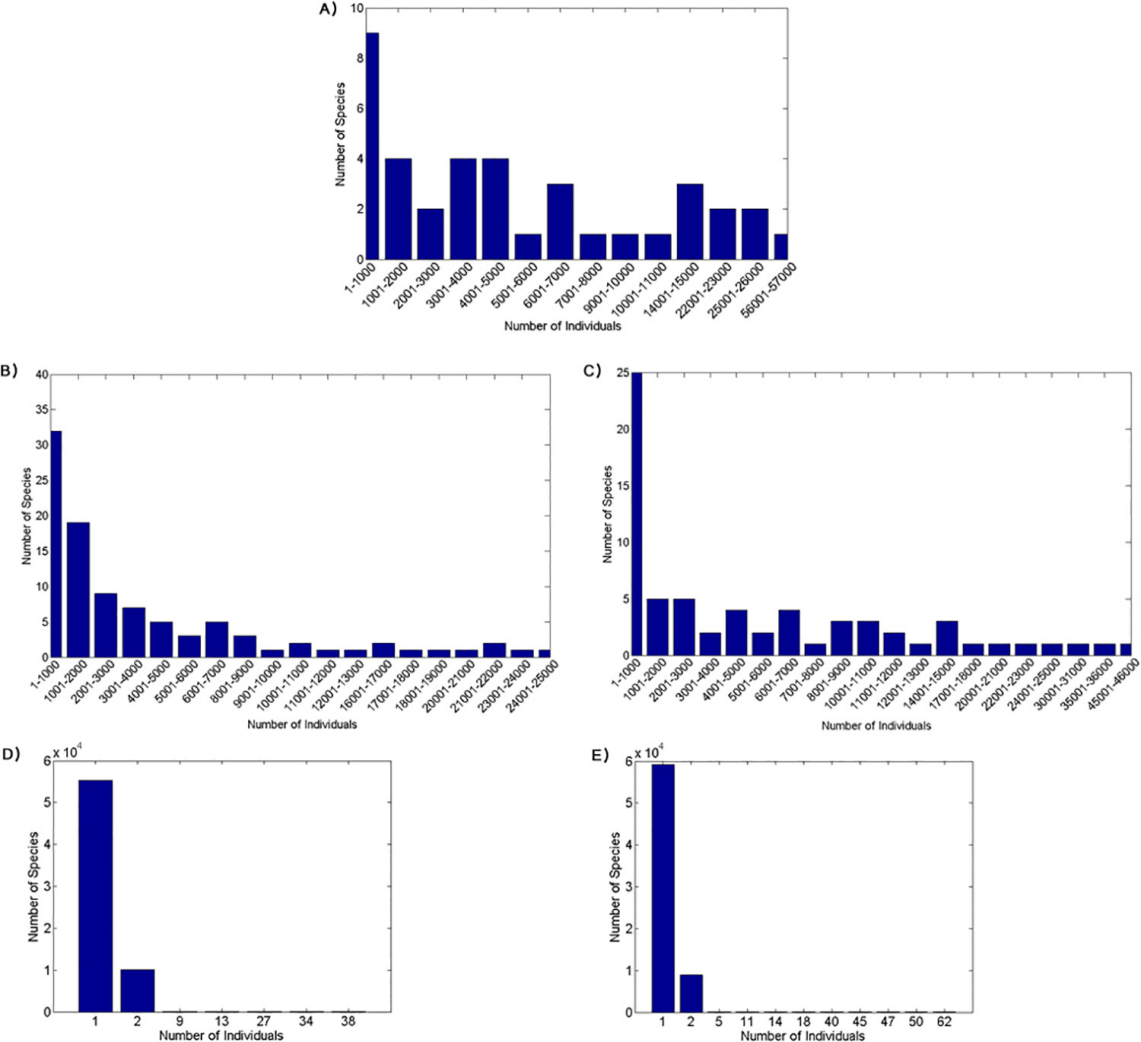
Species abundance distribution in different experiments. (A) Selection, Enforced Reproductive Isolation and Low Dispersal experiment; (B) Selection and Low Dispersal experiment; (C) Selection and High Dispersal experiment; (D)Selection and Low Dispersal experiment; (E) No Selection and High Dispersal experiment.

Moreover, the species abundance distribution patterns observed in the three runs with natural selection follow a Fisher’s logseries (Fig 6). This pattern was also shown in [40]. Many large species (of more than 10,000 individuals) tend to persist for several thousand time steps showing the stability of these genomic clusters. By contrast, the two experiments without natural selection generate a large number of clusters (around 65,000; Table 3) that contain only two or three individuals each. These small clusters tend to persist for only few time steps and have species abundance distribution concentrated in the two first bins (see Fig 6), showing that no organization of genotype groups emerged.

**Fig 7:**
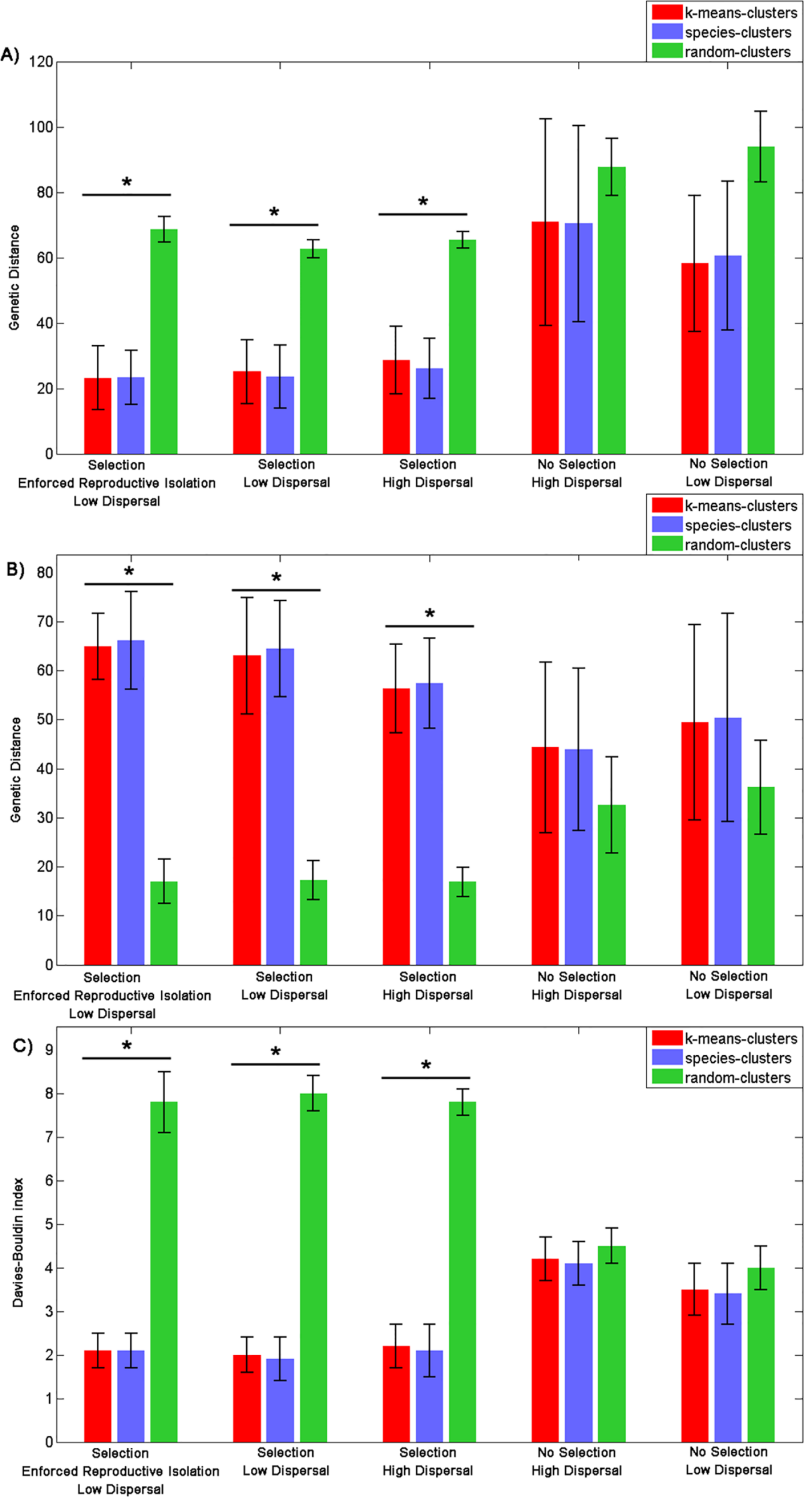
Evaluation of the compactness and separation of clusters. Mean and standard deviation (error bars) of the distance of the farthest individual from its cluster’s genetic centre (**A**), the distance between the genetic centers of all pairs of clusters (**B**) and the Davies-Bouldin index (**C**) for the five experiments. For (**A**) and (**C**) the lower the value the more compact the cluster and the more it is separated from other clusters. For each experiment, the values are given for a global k-means clustering algorithm (blue), the species-clusters generated by the simulation (red) and random clusters (green) (*P<0.05).

Our other speciation metrics support the above assertions: experiments with natural selection led to clusters that were significantly more discrete, in terms of both compactness and separation (genomic distance and the Davies-Bouldin index), than random clusters, whereas experiments without natural selection did not (Fig 7, see section 3–1 in **S1 Materials** for more details). Furthermore, we found no difference in these properties between the **Selection, Enforced Reproductive Isolation and Low Dispersal** experiment, which involves a pre-defined extrinsic mating rule based on genetic distance, and the **Selection and Low Dispersal** experiment (one-way ANOVA for all considered time steps, *P* = 0.6) and the **Selection and High Dispersal** experiment (one-way ANOVA for all considered time steps, *P* = 0.4) (Fig 7) where individuals make free reproductive decisions. This important result reveals the emergence of genetic clusters in the absence of extrinsic (postzygotic) barriers to gene flow but in the presence of natural selection (section 3–1 in **S1 Materials**).

**Fig 8:**
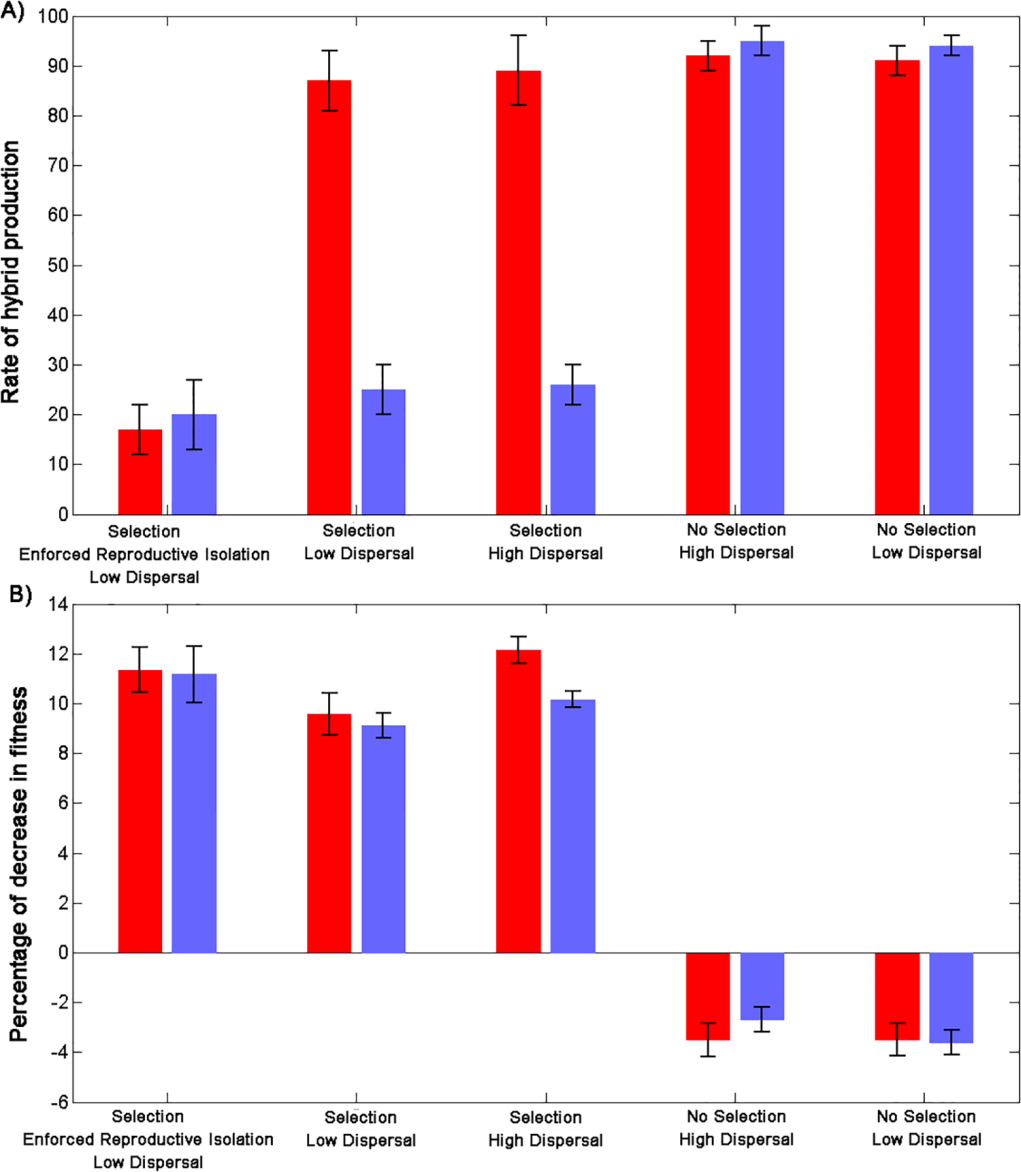
Evaluation of the reproductive barriers between species. (**A**) Mean and standard deviation (error bars) of the rate of hybrid production before (red) and after (blue) 10000 time steps. (**B**) Mean and standard deviation of the percentage of decrease in the fitness of hybrid individuals compared to non-hybrid individuals before (blue) and after (red) 10000 time steps. Fitness values were recorded and averaged every 100 generations.

If the clusters uncovered in our simulations, which correspond to the genotypic cluster concept, also correspond to the biological species concept, then reproductive barriers should be evident between them. We tested for this possibility by quantifying and averaging the rate of hybrid production (Fig 8A) and the fitness of hybrids (Fig 8B) measured every 100 time steps. In the experiments with natural selection, hybridization rate decreased to about 25% after 10,000 generations (i.e., once the number of individuals per species has stabilized, see Fig 5). On average of all cluster species, about 90% of all hybridization events occurred during the first 100 time steps, after two genetic clusters split–that is, hybridization was subsequently uncommon.

**Fig 9:**
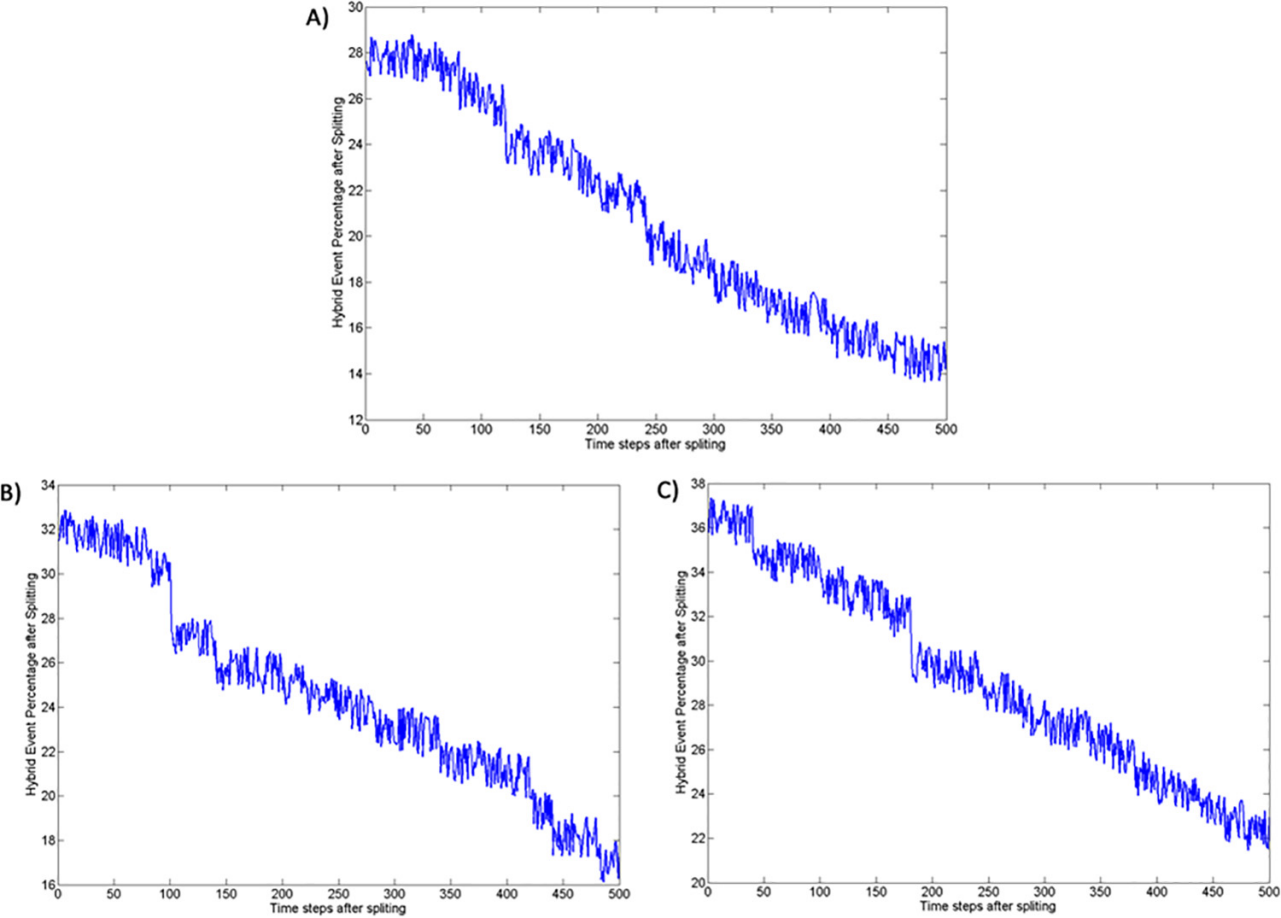
Percentage of hybridization events. (A) Selection, Enforced Reproductive Isolation and Low Dispersal experiment; (B) Selection and Low Dispersal experiment; (C) Selection and High Dispersal experiment.

**Fig 10:**
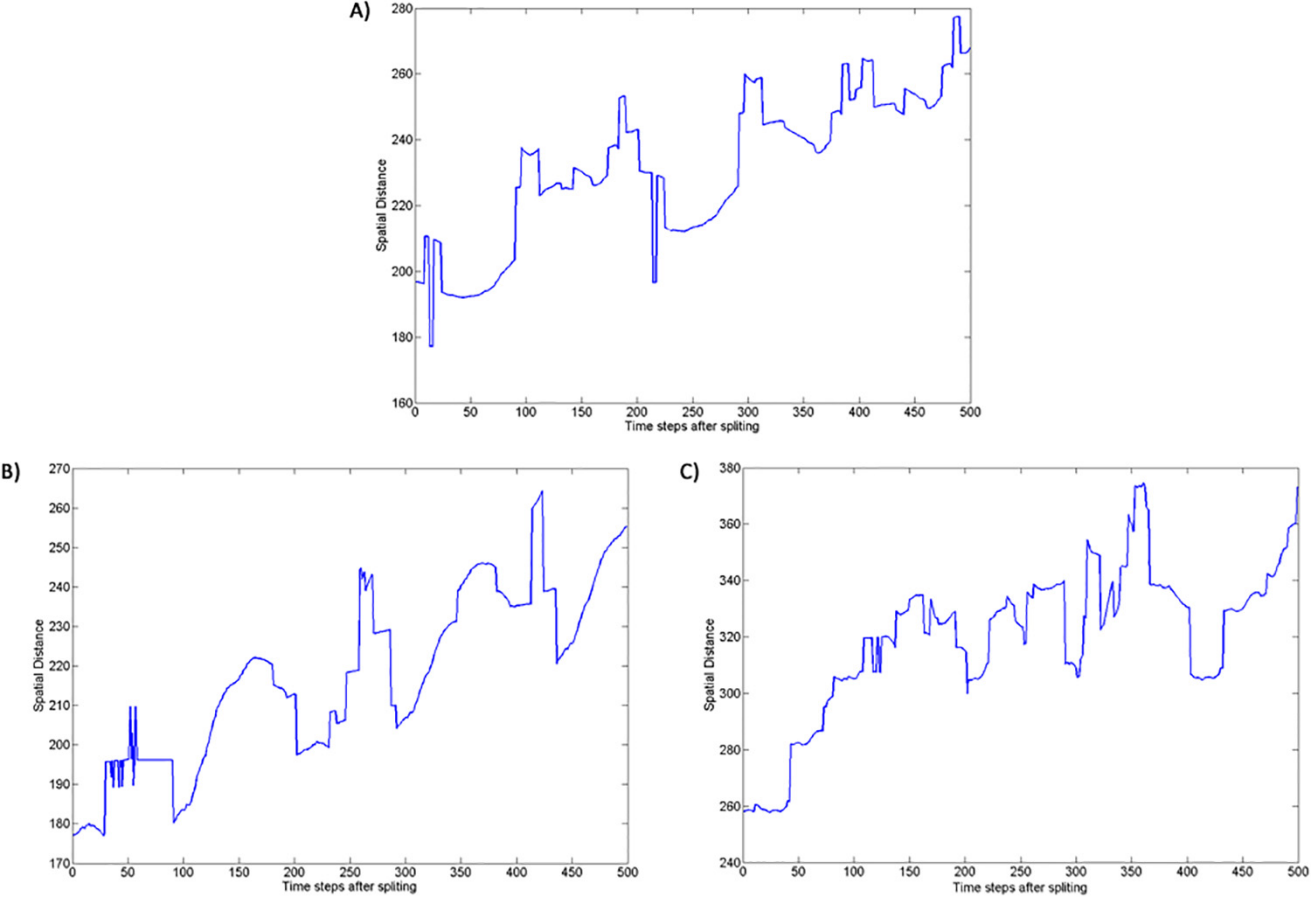
Spatial distance between the sister species. (A) Selection, Enforced Reproductive Isolation and Low Dispersal experiment; (B) Selection and Low Dispersal experiment; (C) Selection and High Dispersal experiment.

**Fig 11:**
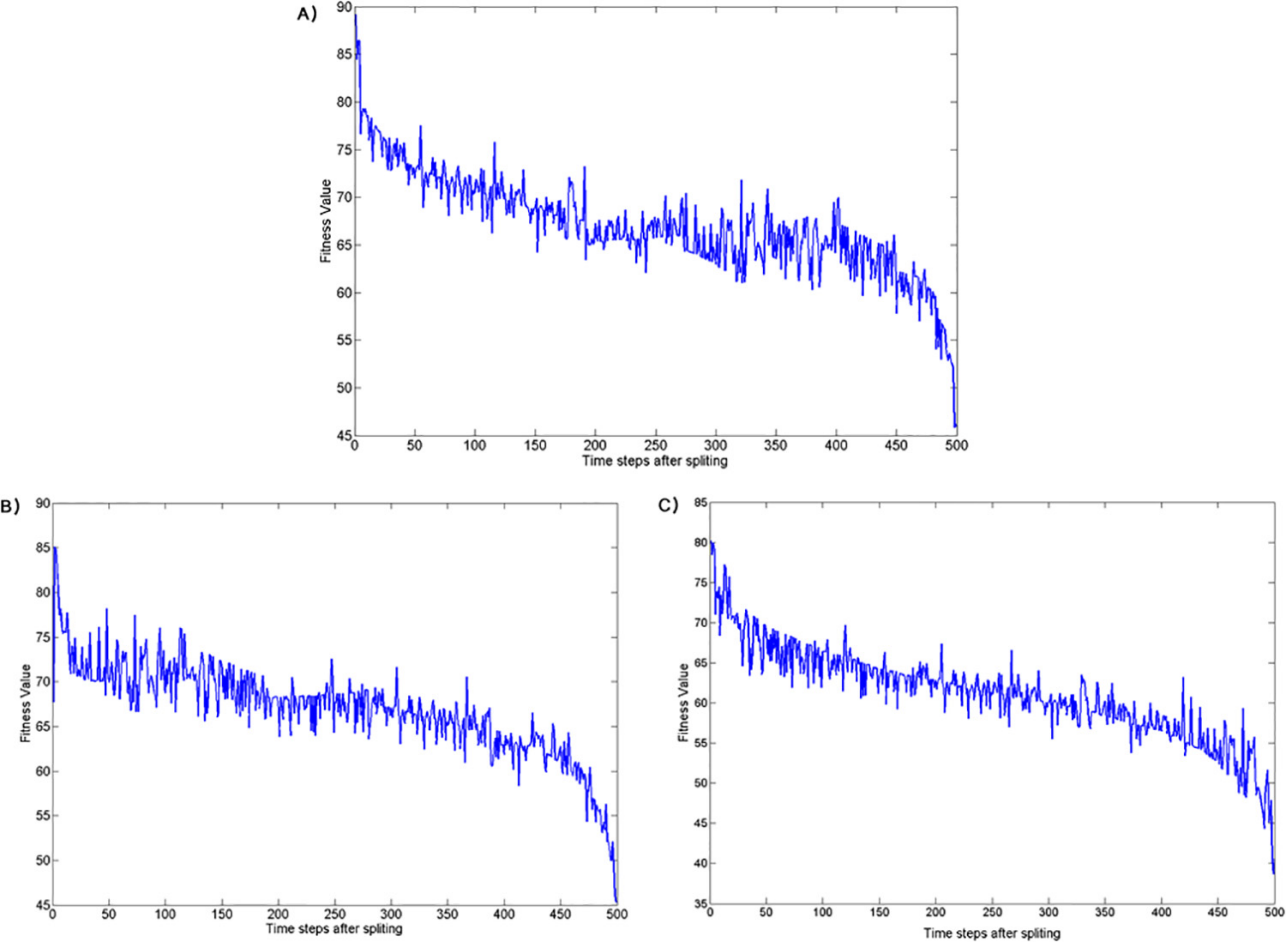
Hybrid fitness between the sister species. (A) Selection, Enforced Reproductive Isolation and Low Dispersal experiment; (B) Selection and Low Dispersal experiment; (C) Selection and High Dispersal experiment.

For the species with long life span and more than 1000 individuals at a given time, the number of hybridization events constantly decrease with time after splitting (Fig 9). Since there is no mate choice, the reduction in the number of hybridization events should be due to an increase in spatial distance between the sister species which increase by about 30% in the first 100 time steps after splitting (see Fig 10). Thus, all simulations that involved selection led to reduced mating between clusters (section 3–2 in **S1 Materials**). In addition, hybrid fitness decreased by about 10–12% in average with a continuous reduction of the fitness of the hybrids during the first 500 time steps after splitting (see Fig 11). And, as before, results for all three selection experiments converged after time step 10,000. Interestingly, hybrid fitness for all selection experiments decreases already before time step 10,000. This steady decrease in hybrid fitness is, in all likelihood, because the species contain a lot of individuals well before reaching time step 10,000 and therefore lead to large sister species. The hybrids generated in these conditions can have highly distant ancestors that are therefore more likely to be strongly differentiated leading to a low fitness offspring. By contrast, similar reproductive barriers were not evident in the simulations without selection (one-way ANOVA, *P* = 0.001; Tukey post hoc test, *P* < 0.05 for all pairs of selection/no selection experiments after time step 10000). These results confirm that the genetic clusters emerging under selection correspond to local fitness maxima, whereas genotypes outside of the clusters have lower fitness. These large compact groups of locally high-fitness genotypes, reproductively isolated from each other, can reasonably be considered as separate species.

In the **Selection, Enforced Reproductive Isolation, and Low Dispersal** experiment, the number of individuals and species stabilized very early (Fig 5). In this experiment, the well-defined reproductive barrier, strong natural selection, and clear spatial isolation between populations act jointly to create a very stable world. As can be expected, this experiment also generated the highest number of individuals per species. In the **Selection and Low Dispersal** experiment, because of the removal of the enforce reproductive isolation mechanism, the number of individuals sharply increase during the first 2000 time steps, then sharply decrease because of an exhaustion of food resources. Then, the number of individuals per species increased steadily and stabilized (Fig 5). In the **Selection and High Dispersal** experiment (Fig 5), the number of individuals per species was initially lower than in the **Selection and Low Dispersal** experiment and then much higher from time step 3000 to 10,000, during the recovery period, after exhaustion of the food resources. In the high dispersal configuration many isolated sub-populations are formed reducing the overall gene flow leading to a higher number of species. Subsequently, the number of individuals per species of the **Selection and High Dispersal** experiment finally converged to the same value as the two other experiments with selection. These phenomena may be explained by the competition for resources between individuals. The increase in genetic diversity due to genetic drift and large population sizes is likely countered by natural selection, as the individuals too genetically different from their parents will not benefit from their co-adapted gene complexes and, thus, behavioral suites. This finding was confirmed by the lower fitness for the hybrid individuals when compared to no-hybrid parental species (section 3–2 in **S1 Materials**).

In the experiments without natural selection, in contrast, genetic diversity (and, hence, the number of species) simply increased until each species was represented by only a few individuals (Fig 5). In these experiments, the individuals do not use a behavioral model to perceive the world and chose their action. Further, there is no competition between individuals for resources because birth and death was only governed by the Lotka-Volterra model. As a result, genetic diversity increases with time and is not counteracted by the filtering of natural selection, leading to the emergence of increasing numbers of species. However, these ‘species’ do not represent pools of similar genomes, well separated from the others and do not adhere to the genomic cluster or biological species concepts (section 3–1 in **S1 Materials**)–so they aren’t really species.

De Aguiar [38], using a model similar to our **No Selection and Low Dispersal** model but with **Enforced Reproductive Isolation**, found the emergence of species for some configurations of the parameter of their system. However, the results they present cover only 1000 generations and unfortunately, the variation of the number of species with time was not tracked. It is likely that the number of species would continue to increase if the simulation is run for longer time leading to the same observations we obtained with our experiment without selection, that is a continuous increase in the number of species until each species contain only two or three individuals.

The role of natural selection in the formation of divergent behavioural models clearly interacted with the role of spatial structure. In particular, in the experiments with natural selection and low-dispersal, species tended to be strongly spatially clustered, with 95% of the individuals of a newly-formed species occupying a number of cells that represent an area that is 2.5–10% of the whole world, even though these cells can be spread at different places of the world. This is presumably because spatially localized clusters more easily maintain cohesiveness across the entire cluster and less frequently encounter other clusters (promoting genotypic divergence between clusters). This spatial clustering was stronger at low than at high dispersal (Table D in **S1 Materials**), it decreases with the life span of the species, and divergent genotypic clusters arose correspondingly more quickly under the former than the latter. Eventually, however, the number of species in the **Selection and High Dispersal** experiment converged on that for the **Selection and Low Dispersal** experiment, confirming that as many species can arise under high dispersal as under low dispersal–it just takes longer.

## Conclusion

Hundreds of mathematical models have been developed to study the role of selection in speciation [2–4], and the general view to have emerged is that selection causes speciation under a specific subset of conditions. These previous models used pre-defined functions (e.g. for competition, carrying capacity, overall fitness etc.) that leave open the question of whether or not the findings are particular to those functions. Our model did not include such functions and instead allowed selection to emerge as a result of complex behavioural interactions. Under these conditions, speciation occurred in different configurations with selection but not without selection, thus providing further support for the role of selection in driving speciation [41, 42].

In our model, speciation occurred due to biotic interactions, both within and between species. Whereas there is no evolution of specific traits modelling an “arm race”, the evolution is still driven by the behavioural model. These biotic interactions drove the evolution of a diversity of behavioural types, and these different types formed discrete genotypic (and often spatial) clusters. Mating between these emerging clusters rapidly decreased, and hybrids between then soon had low fitness. Although abiotic conditions can certainly drive speciation, our results support assertions that biotic interactions could be particularly important drivers of the selection that causes the formation of new species [41–43]. Importantly, given the uniformity of resource production, our model is not a model of ecological speciation in the typical sense [42]. While spatial divergence in predators or prey could certainly lead to spatially divergent selection for different behaviors, it seems likely that many new species simply possessed alternative behavioural solutions for similar environments, with those solutions being incompatible with each other–i.e., a sort of ‘mutation-order’ speciation [10], in which incompatible mutations led to divergent behavioural models that all sought to acquire the same resources and avoid the same predators.

Although speciation can be driven by morphological or physiological divergence, our results support arguments that speciation might proceed particularly rapidly as a result of behavioural divergence [44, 45]. Other forms of behaviour, such as sexual selection, can inhibit (or promote) speciation [46], and it would be interesting to combine these aspects into a single model. Of course, our model–like all previous models–is still a gross simplification of nature. So the next important step is to develop testable predictions that can be used to evaluate the extent to which model assumptions and outcomes are predictive of the natural world. For instance, an interesting starting point with respect to our model would be to develop detailed ethograms for the behaviours of closely related species or diverging populations. Divergence in these behavioural repertoires could then be examined for their likely contribution to limiting gene flow between populations by creating unfit hybrids, whether in similar or different environments.

## Supporting Information

S1 Fig Spatial distribution of individuals in the different versions of simulation. (**A**) Selection, Enforced Reproductive Isolation, and Low Dispersal experiment (**B**) Selection and Low Dispersal experiment (**C**) Selection and High Dispersal experiment (**D**) No Selection and Low Dispersal experiment (**E**) No Selection and High Dispersal experiment. Different colors stand for different prey species. Predators are represented in white.
(TIF)

S1 Materials Presenting the general considerations of predefined fitness function in ecosystem modeling, extended material and methods, experimental design and results. (DOC)
