# Supplementary figures and images for: Speciation without Pre-Defined Fitness Functions
Source: PLoS One. 2015 Sep 15;10(9):e0137838. doi: 10.1371/journal.pone.0137838 (PMC4570812; doi:10.1371/journal.pone.0137838)

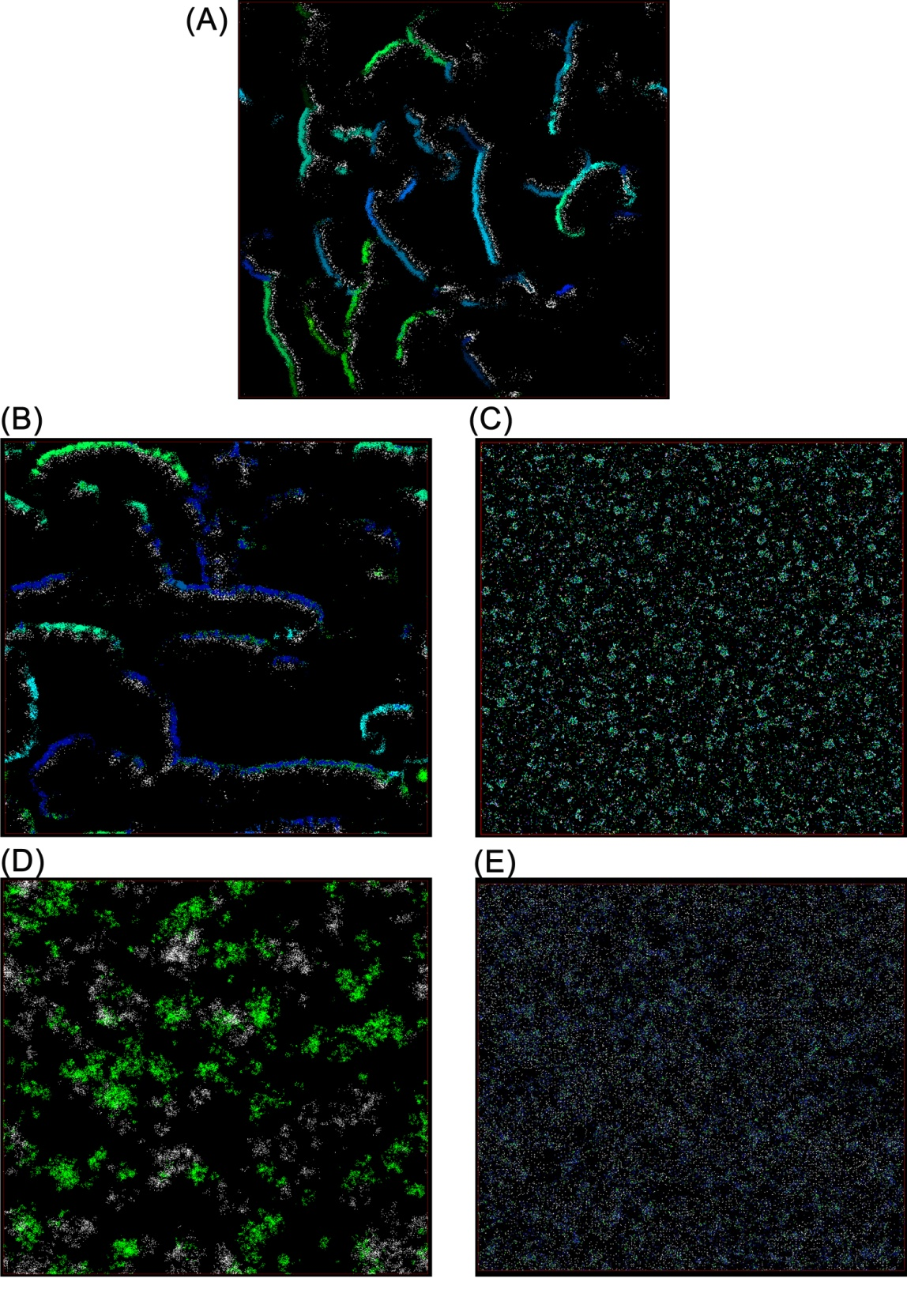

Supplement: S1 Fig — (A) Selection, Enforced Reproductive Isolation, and Low Dispersal experiment (B) Selection and Low Dispersal experiment (C) Selection and High Dispersal experiment (D) No Selection and Low Dispersal experiment (E) No Selection and High Dispersal experiment. Different colors stand for different prey species. Predators are represented in white. (TIF) [file pone.0137838.s001.tif]
