# Supplementary material for: Speciation without Pre-Defined Fitness Functions
Source: PLoS One. 2015 Sep 15;10(9):e0137838. doi: 10.1371/journal.pone.0137838 (PMC4570812; doi:10.1371/journal.pone.0137838)
Supplement: S1 Materials — (DOC) [file pone.0137838.s002.doc]

Supplementary Information for

Speciation without pre-defined fitness functions

Robin Gras^1,2,3^, Abbas Golestani^1^, Andrew P. Hendry^4^, Melania Cristescu^4^

^1^School of Computer Science, University of Windsor, Windsor, ON, Canada.

^2^Department of Biology, University of Windsor, Windsor, ON, Canada.

^3^Great Lakes Institute for Environmental Research, Windsor, ON, Canada.

^4^Redpath Museum & Department of Biology, McGill University, Montreal, QC, Canada

1. General Considerations: Predefined fitness function

Artificial evolving systems with pre-defined fitness functions, or fitness landscapes, have been well studied. In the 1960s, John Holland introduced Genetic Algorithm (GA) [1] as a tool to model the adaptation of organisms to their environment and to develop ways in which complex evolutionary processes can be investigated in computer systems [2]. Since then, many empirical and theoretical studies have been undertaken to determine the behavior of such artificially evolving systems [3].

To model evolutionary systems, fitness is assigned to individuals based on some genetic or phenotypic properties associated with the individual. In general, the fitness function is an *a priori* feature of the model, leading to what Packard called extrinsic adaptation [4] and Channon et al. called artificial selection [5]. The dynamic behavior of such a system (exploring the set of all possible population (set) of genomes) is well understood: the population converges to the peaks of the fitness function [3]. Systems such as Genetic Algorithms with fitness sharing (also called niching) allow the modeling of competition for resources and can generate population distributions not centered on the peaks [6, 7]. The behaviors of such approaches are also well known to show convergence, with possible cycles, towards the peaks of the fitness function modified by the fitness sharing process. This is similar to the experiment performed by Dieckmann and Doebeli [16] with a high mutation rate that forces the exploration of all the peaks. Problems in which the fitness function varies through time have also been studied, showing that the population distribution converges towards multiple successive points, each one linked to the peaks of the new version of the fitness function [8]. Other work has studied the effect of linkage between loci (also called ‘dependency’, see the review by Chen [9]), which leads to a new type of genetic algorithm called Estimation Distribution Algorithm [10] that can also considers the hierarchy of linkages [11]. More complex systems based on artificial selection were designed and discussed in [5, 12].

All of these systems are optimization processes, even with fitness sharing where they do not converge toward a simple “mean fitness” but toward equilibrium of species distributed among the different peaks. It means that the fate of the system is directly determined by its pre-defined fitness function with the convergence behavior described above. Packard was the first to design a simple model not governed by extrinsic adaptation, which demonstrated that the evolutionary dynamic could be an emerging property of an intrinsic model [4, 13]. Unfortunately, their organisms and systems were too simple for new species to emerge. Moreover, the more complex systems, such as Geb [12] and Polyworld [14], rely heavily on learning very complex neural networks to model behavior, and the associated computational requirements constrain the total population to a few hundred individuals for a few hundred generations. Such models are thus inefficient in dealing with processes, such as speciation, that can span large spatial and temporal scales.

To our knowledge, all previous speciation models rely on one form or another of an *a priori* fitness function, that is a function that assigns directly or indirectly fitness to one or several genomic traits. This includes frequency-dependent selection as it is a fitness sharing process and therefore an optimization process. Since it is obviously true for all purely mathematical, we here focus our discussion on individual-based models (IBMs), providing a few representative examples. In these studies, the pre-defined fitness function is generally defined as a fitness landscape [15], which is a classical representation also used to study the properties of Genetic Algorithms.

- Gavrilets proposed a simple model in which L loci are each assigned a binary fitness value (*fit* or *unfit)*, later extended to a continuous range of fitness [15]. As these fitness values are initially set and do not evolve during the simulation, the fitness landscape is predefined.
- Gavrilets [16] used an IBM approach in a bidimensional environment with two types of cells containing different resources. The fitness of an individual is modeled by two Gaussian functions with pre-defined parameters corresponding to the two types of cells, which generate a fixed multimodal fitness landscape.
- Dieckmann [17], Kirkpatrick [18] and Bolnick [19] used a Gaussian fitness function coupled with a Gaussian competition function similar to the fitness sharing model analyzed in Genetic Algorithm with niching. Both functions use fixed pre-defined parameters generating a landscape where the peaks are fixed. As said above, these systems do not converge towards a mean fitness but generate an equilibrium (possibly an oscillating equilibrium) of species distributed among the peaks.
- Drossel [20] and Doebeli [21] associated an IBM with Lotka-Volterra competition equations, similar to [17-19], that predefined phenotypic fitness. Doebeli [22] designed an IBM in a bidimensional environment in which the fitness of individuals is governed by a Lotka-Volterra model with a fixed fitness landscape, composed of a succession of peaks, defined by a linear gradient of resources and associated with fitness sharing based on genomic similarity.
- Higashi [23] proposed a model where an *a priori* fitness function is computed on L additive loci. This model studies the possibility of sympatric speciation by two simultaneous processes of run-away sexual-selection.
- Takimoto [24] proposed a model, based on the sexual-selection model presented in [23], with an *a priori* fitness function computed on a deterministic combination of the alleles of three loci.
- Gravilets [25, 26] and Thibert-Plante [27, 28] defined IBMs based on pre-defined multimodal Gaussian distributions of resources associated with a normalized competition function. Even though the resulting dynamic of such probabilistic complex systems can lead to non-stationary and non-converged population distributions, their overall behaviors are pre-determined and can be studied as in Débarre [29].
- The approaches based on individual-based evolutionary game models (IBEG models; see the review of Allen [30]) integrate complex competition models but are still based on a pre-defined fitness function because of the pre-defined pay-off function.
- Metz [31, 32] presented methods based on a matrix of probabilities to describe the variations of the states of an IBM system and can be used to derivate the evolution of these states. These approaches do not rely on a pre-defined fitness function. However, in addition to being restricted to clonal populations, even if it seems that Mendelian populations could be approximated with some restrictions, they rely on the construction of transition tables of incommensurable size associated to intractable computations as soon as the system model is not extremely small. These approaches are therefore not practicable as IBM simulations of complex ecosystems.

Relying on pre-defined fitness functions, all of these methods correspond to one form or another of a genetic algorithm and they perform an optimization process with predictable convergence properties. The only models not relying on pre-defined fitness functions lead to intractable computations to study even very simple systems. We suggest that, for this reason, previous studies did not allow to study the emergence of intrinsic adaptations in the sense of Packard [4].

In EcoSim, all the factors determining the reproductive success of an individual are free of pre-defined fitness functions. The overall fitness of an individual, measured as its reproductive success and that of its offspring, depends only on the interaction between its phenotype (behavioral type that is free to evolve) and the environment. At each time step, the individuals in EcoSim consume some energy. This energy consumption is determined by a cost function that takes into account the complexity of the behavioral model of the individual (the number of edges it contains) and the action it performs. The more complex the model and the faster the movements performed by the individual (such as escape and exploration), the more energy is used. This cost function is pre-defined. Nevertheless, a cost function is not equivalent to a fitness function, since it represents merely the metabolism of the individual and can be seen as a ‘fix penalty’ which is assigned to behavioral models and actions independently of the environment in order to avoid an obvious continuous increase in the model complexity and to model energy depletion with time. The success of a behavioral model relies on the trade-off between the decisions it makes, knowing the current environment, and the cost of the actions it performs throughout its life. However, this trade-off is not arbitrated by a predefined extrinsic function but results from the consequence of the actions undertaken.

As a consequence, decisions made by individuals with distinct behavioral models do not rely on any external evaluation (pre-defined fitness function). Instead, decisions rely on the knowledge ‘learned’ from the environment in the behavioral model by the evolutionary process, tuning behaviors to a particular state of the local world, and on the individual perception of the local environment. The model determining the reproductive success of an individual is thus intrinsic to the simulation in the sense that no external information is involved for determining fitness.

**2- Extended Materials and Methods**

**2-1 Submodels in EcoSim**

Each individual performs one unique action during a time step based on its perception of the environment. EcoSim iterates continuously, and each time step consists of the computation of the activation level of the nodes using the FCM of each individual (the initial values of the edges of the FCM are given in Table A for prey and Table C for predators), leading to the choice and application of an action for every individual. A time step also includes the update of the world: emergence and extinction of species and growth and diffusion of grass, or decay of meat.

Table A. Initial FCM values for prey (See Table B). Every prey individual has a FCM which represents its behaviour. At first time step, all prey individuals have an initial FCM. During time, with operators like crossover and mutations, the FCMs of individuals evolve.

|  | **FR** | **HG** | **SP** | **CU** | **SD** | **ST** | **NU** | **ES** | **SF** | **SC** | **XP** | **WT** | **ET** | **RP** |
| --- | --- | --- | --- | --- | --- | --- | --- | --- | --- | --- | --- | --- | --- | --- |
| **PC** | 4 | 0 | 0 | 0.1 | 0 | -1 | 1 | 0 | 0 | 0 | 0 | 0 | 0 | 0 |
| **PF** | -4 | 0 | 0 | 0 | 0 | 0.5 | -0.5 | 0 | 0 | 0 | 0 | 0 | 0 | 0 |
| **OC** | 0 | 0.5 | 0 | -0.1 | 0.1 | 0.5 | -0.5 | 0 | 0 | 0 | 0 | 0 | 0 | 0 |
| **OF** | 0 | 0 | -0.4 | 0.2 | -0.2 | -0.7 | 0.7 | 0 | 0 | 0 | 0 | 0 | 0 | 0 |
| **FC** | 0 | 0 | 0.5 | -0.1 | 0.1 | 0.5 | -0.5 | 0 | 0 | 0 | 0 | 0 | 0 | 0 |
| **FF** | 0 | 0 | -0.4 | 0.2 | -0.2 | -0.5 | 0.5 | 0 | 0 | 0 | 0 | 0 | 0 | 0 |
| **EL** | 0.4 | 4 | -1.5 | 0 | 0 | -2.2 | 2.2 | 0 | 0 | 0 | 0 | 0 | 0 | 0 |
| **EH** | 0 | -1 | 1.5 | 0.2 | -0.2 | 1.5 | -1.5 | 0 | 0 | 0 | 0 | 0 | 0 | 0 |
| **OH** | 0 | -0.2 | 0 | -0.3 | 0.3 | 1.1 | -1.1 | 0 | 0 | 0 | 0 | 0 | 2.6 | 0 |
| **OL** | 0 | 0.2 | 0 | 1 | -1 | -1.1 | 1.1 | 0 | 0 | 0 | 0 | 0 | -4 | 0 |
| **PY** | 0 | 0 | 0 | -0.4 | 0.4 | 0.5 | -0.5 | 0 | 0 | 0 | 0 | 0 | 0 | 1.5 |
| **PN** | 0 | 0 | 0.5 | 0.3 | -0.3 | -0.8 | 0.8 | 0 | 0 | 0 | 0 | 0 | 0 | -4 |
| **FR** | 0.5 | 0 | 0 | 0 | 0 | 0 | 0 | 1.5 | -0.8 | -1 | 0.3 | -1 | -1 | -1 |
| **HG** | 0 | 0.3 | 0 | 0 | 0 | 0 | 0 | -0.8 | 2.1 | -0.7 | 0.7 | -0.5 | 4 | -1.8 |
| **SP** | 0 | 0 | 0.2 | 0 | 0 | 0 | 0 | -0.2 | 0 | 1.5 | 0.5 | -0.3 | -0.4 | 3 |
| **CU** | 0 | 0 | 0 | 0.1 | 0 | 0 | 0 | -0.1 | 0.5 | 0.3 | 1.5 | -0.2 | -0.3 | -0.2 |
| **SD** | 0 | 0 | 0 | 0 | 0.1 | 0 | 0 | 0 | -0.5 | -0.3 | -1.2 | 0.2 | 0.3 | 0.2 |
| **ST** | 0 | 0 | 0 | 0 | 0 | 0 | 0 | -0.1 | -0.8 | -0.2 | -2 | 1.5 | 0.8 | 0.7 |
| **NU** | 0 | 0 | 0 | 0 | 0 | 0 | 0 | 0.4 | 1 | 0.2 | 2 | -1.2 | -0.7 | -0.7 |
| **ES** | 0 | 0 | 0 | 0 | 0 | 0 | 0 | 0 | 0 | 0 | 0 | 0 | 0 | 0 |
| **SF** | 0 | 0 | 0 | 0 | 0 | 0 | 0 | 0 | 0 | 0 | 0 | 0 | 0 | 0 |
| **SC** | 0 | 0 | 0 | 0 | 0 | 0 | 0 | 0 | 0 | 0 | 0 | 0 | 0 | 0 |
| **XP** | 0 | 0 | 0 | 0 | 0 | 0 | 0 | 0 | 0 | 0 | 0 | 0 | 0 | 0 |
| **WT** | 0 | 0 | 0 | 0 | 0 | 0 | 0 | 0 | 0 | 0 | 0 | 0.2 | 0 | 0 |
| **ET** | 0 | 0 | 0 | 0 | 0 | 0 | 0 | 0 | 0 | 0 | 0 | 0 | 0 | 0 |
| **RP** | 0 | 0 | 0 | 0 | 0 | 0 | 0 | 0 | 0 | 0 | 0 | 0 | 0 | 0 |

Table B. Prey/predator FCM abbreviation table. The abbreviation used to present concepts of FCM in EcoSim. These abbreviations have been used in other tables to show values of these concepts.

| **NodeName** | **Abbreviation** | **NodeName** | **Abbreviation** |
| --- | --- | --- | --- |
| Fear | FR | PredClose | PC |
| Hunger | HG | PredFar | PF |
| SearchPartner | SP | FoodClose | OC |
| CuriosityStrong | CU | FoodFar | OF |
| Sedentary | SD | FriendClose | FC |
| Satisfaction | ST | FriendFar | FF |
| Nuisance | NU | EnergyLow | EL |
| Escape | ES | EnergyHigh | EH |
| SearchFood | SF | FoodLocalHigh | OH |
| Socialize | SC | FoodLocalLow | OL |
| Exploration | XP | PartnerLocalYes | PY |
| Wait | WT | PartnerLocalNo | PN |
| Eat | ET | PreyClose | YC |
| Reproduce | RP | PreyFar | YF |
| ChaseAway | CA |  |  |
| SearchPrey | SY |  |  |

Table C. Initial FCM values for predator (See Table B). Every predator individual has a FCM which represents its behaviour. At first time step, all predator individuals have an initial FCM. During time, with operators like crossover and mutation, the FCM of individuals change

|  | **CA** | **HG** | **SP** | **CU** | **SD** | **ST** | **NU** | **SY** | **SF** | **SC** | **XP** | **WT** | **ET** | **RP** |
| --- | --- | --- | --- | --- | --- | --- | --- | --- | --- | --- | --- | --- | --- | --- |
| **YC** | 0.7 | 0 | 0 | -0.1 | 0 | 0.5 | -0.5 | 0 | 0 | 0 | 0 | 0 | 0 | 0 |
| **YF** | -0.5 | 0.7 | 0.1 | 0.4 | -0.4 | -0.5 | 0.5 | 0 | 0 | 0 | 0 | 0 | 0 | 0 |
| **OC** | -0.5 | 0.7 | 0 | -0.1 | 0.1 | 0.5 | -0.5 | 0 | 0 | 0 | 0 | 0 | 0 | 0 |
| **OF** | 0.8 | -0.2 | 0.1 | 0.2 | -0.2 | -0.6 | 0.6 | 0 | 0 | 0 | 0 | 0 | 0 | 0 |
| **FC** | 0 | 0 | 0.7 | 0 | 0 | 0.4 | -0.4 | 0 | 0 | 0 | 0 | 0 | 0 | 0 |
| **FF** | 0 | 0 | -0.5 | 0.3 | -0.3 | -0.4 | 0.4 | 0 | 0 | 0 | 0 | 0 | 0 | 0 |
| **EL** | 3.5 | 5 | -1.2 | 0 | 0.2 | -1.5 | 1.5 | 0 | 0 | 0 | 0 | 0 | 0 | 0 |
| **EH** | -2 | -3 | 1.4 | 0.3 | -0.3 | 1 | -1 | 0 | 0 | 0 | 0 | 0 | 0 | 0 |
| **OH** | -1.5 | 0.3 | -0.2 | -0.3 | 0.3 | 1 | -1 | 0 | 0 | 0 | 0 | 0 | 4 | 0 |
| **OL** | 1.7 | 0 | 0.2 | 1 | -1 | -1 | 1 | 0 | 0 | 0 | 0 | 0 | -5 | 0 |
| **PY** | -0.3 | 0 | 0 | -0.4 | 0.4 | 0.8 | -0.8 | 0 | 0 | 0 | 0 | 0 | 0 | 2 |
| **PN** | 0.3 | 0 | 0.5 | 0.3 | -0.3 | -0.8 | 0.8 | 0 | 0 | 0 | 0 | 0 | 0 | -5 |
| **CA** | 0.2 | 0 | 0 | 0 | 0 | 0 | 0 | 1.5 | -0.2 | -0.4 | 0.3 | -0.4 | 0 | -0.4 |
| **HG** | 0 | 0.3 | 0 | 0 | 0 | 0 | 0 | 4 | 2.5 | -1.2 | 0.3 | -0.4 | 3.5 | -0.8 |
| **SP** | 0 | 0 | 0.2 | 0 | 0 | 0 | 0 | -0.8 | -0.8 | 1.5 | 0.3 | -0.5 | -0.6 | 3 |
| **CU** | 0 | 0 | 0 | 0.1 | 0 | 0 | 0 | 0.3 | 0.3 | 0.3 | 1.5 | -0.4 | -0.3 | -0.2 |
| **SD** | 0 | 0 | 0 | 0 | 0.1 | 0 | 0 | -0.3 | -0.3 | -0.3 | -1.5 | 0.4 | 0.3 | 0.2 |
| **ST** | 0 | 0 | 0 | 0 | 0 | 0 | 0 | -0.8 | -0.8 | -0.2 | -1.8 | 1 | 0.8 | 0.8 |
| **NU** | 0 | 0 | 0 | 0 | 0 | 0 | 0 | 1 | 0.8 | 0.2 | 2 | -1 | -0.6 | -0.8 |
| **SY** | 0 | 0 | 0 | 0 | 0 | 0 | 0 | 0 | 0 | 0 | 0 | 0 | 0 | 0 |
| **SF** | 0 | 0 | 0 | 0 | 0 | 0 | 0 | 0 | 0 | 0 | 0 | 0 | 0 | 0 |
| **SC** | 0 | 0 | 0 | 0 | 0 | 0 | 0 | 0 | 0 | 0 | 0 | 0 | 0 | 0 |
| **XP** | 0 | 0 | 0 | 0 | 0 | 0 | 0 | 0 | 0 | 0 | 0 | 0 | 0 | 0 |
| **WT** | 0 | 0 | 0 | 0 | 0 | 0 | 0 | 0 | 0 | 0 | 0 | 0.2 | 0 | 0 |
| **ET** | 0 | 0 | 0 | 0 | 0 | 0 | 0 | 0 | 0 | 0 | 0 | 0 | 0 | 0 |
| **RP** | 0 | 0 | 0 | 0 | 0 | 0 | 0 | 0 | 0 | 0 | 0 | 0 | 0 | 0 |

At initialization time there is no meat in the world, and grass units are distributed randomly. For each cell, there is a probability, probaGrass, that the initial number of grass units is strictly greater than 0. In this case, the initial number is generated uniformly between 1 and maxGrass. Each unit of food provides a fixed amount of energy to the agent that eats it. The prey can only eat grass, and the predators have two modes of foraging: hunting and scavenging. When a predatorʼs hunting action succeeds, a new meat unit is added to the corresponding cell, and the predator is considered to consume another one. When a predatorʼs scavenging action succeeds, one unit of meat is removed from the corresponding cell. When a prey dies, the number of meat units in its cell increases by 2. The number of grass units in a cell decreases by 1 when a prey eats, and the number of meat units decreases by 1 when a predator eats. The number of meat units in a cell also decreases at each time step by one unit due to decay, even if no meat has been eaten.

For each cell of the world, if its number of grass units is greater than zero, half a unit is added per time step; else if one of its eight adjacent cells contains grass, the same number of units is added with probability probaGrowGrass. With this mechanism, if the prey eats all the grass in one cell, the grass cannot regrow unless there still is grass in an adjacent cell. This models the problem of overexploitation of resources and the diffusion of resources through the world.

Evasion (for prey only). The evasion direction is the direction opposite to the direction of the barycenter of the 5 closets enemies within the vision range of the prey, with respect to its current position. If no predator is within the vision range of the prey, the direction is chosen randomly. The new position of the prey is computed using its speed direction and the current activation level of fear is divided by 2.

Hunting (for Predator only). The predator selects the closest cell in its range of vision (including its current cell) that contains at least one prey and moves towards that cell at its current speed. If it reaches the cell, it kills one randomly chosen prey, eating one unit and having another unit of food added to the cell. If the speed of the predator is not enough to reach the prey, it moves at its speed toward this prey. If the predator does not have enough energy to reach to the prey or its speed is not sufficient, the hunting action fails but a movement is performed in direction of the prey.

Search for food. The direction toward the closest food (grass or meat but not leaving prey) within the vision range is computed. If the speed of the agent is high enough to reach the food, the agent is placed on the cell containing this food. Otherwise, the agent moves at its speed toward this food.

Socialization. The direction toward the closest possible mate within the vision range is computed. If the speed of the agent is high enough to reach the mate, the agent is placed on the cell containing the mate but no reproduction action is performed, and the current activation level of sexual needs is divided by 3. Otherwise, the agent moves at its speed toward the mate. If no possible mate is within the vision range of the agent, the direction is chosen randomly.

Exploration. The direction is computed randomly. The agent moves at its speed in this direction. The activation level of curiosity is divided by 1.5.

Resting. No action is taken.

Eating. If the current number of grass (or meat) units is greater than 1, then this number is decreased by 1 and the preyʼs (predatorʼs) energy level is increased by energyGrass (energyMeat). Its activation level for hunger is divided by 4. Otherwise nothing happens.

Breeding.The process of generating a new offspring consists of the following steps. First, the conditions for successful mating are checked. Second, the value of birthEnergyPrey is transmitted (with possible mutations) from one randomly chosen parent to the offspring. Third, the edges’ values are transmitted with possible mutations, and the initial energy of the offspring is computed. To model the crossover mechanism, the edges are transmitted by block from one parent to the offspring. For each node, its outgoing edges’ values are transmitted together from the same randomly chosen parent. Fourth, the maximum age of the offspring is computed. Finally, the energy level of the two parents is updated.

Our speciation method, for a given species S, begins by finding the individual with the greatest Manhattan distance from the species’ center (a vector containing the average of the gene values of its members). If this distance is greater than the predefined threshold for speciation, 2-means clustering is performed. Otherwise, species *S* remains unchanged. If clustering is to be performed, two new species are created – one centered around a random individual in *S*, denoted *I_r_*, and another centered around the individual in S that is farthest from *I_r_*, denoted *I_f_*. Subsequently, all remaining individuals in *S* are added to one of the two new species – whichever species the individual is more genetically similar to. After recalculating the centers for the two new species, this clustering process is repeated until convergence.

After the 2-means clustering is completed, there are two new species, *S_1_* and *S_2_*, whose members are subsets of the original members of *S*. The species closer to the original species *S* inherits the properties of *S*, such as the species ID and the ID of its parent species. Thus, one of the new species will continue to represent the original species, while the other one will represent a split-off of the original species.

**2-2- Measure of spatiotemporal distribution and Experimental design**

We used the Spatiotemporal Complexity (STC) measure to obtain a quantitative comparison of the level of grouping between individuals. This measure has been developed for the analysis of individual patches and is typically used to measure the dispersion or “clumpiness” of different patches of individuals [32]. Values close to ‘one’ correspond to a random uniform distribution of individuals in the world, and values close to ‘zero’ correspond to a unique group of individuals. For each experiment, we conducted ten independent runs using the same physical characteristics (see Table D).

**Table D.** **Several physical and life history characteristics of individuals averaged over 10 independent runs for each experiment.** Exp1 stands for **Selection, Enforced Reproductive Isolation, and Low Dispersal**, Exp2 for **Selection and Low Dispersal**, Exp3 for **Selection and High Dispersal**, Exp4 for **No Selection and High Dispersal** and Exp5 for **No Selection and Low Dispersal**. In the experiments without natural selection, because there is no behavioral model, some characteristics do not exist.

| **Characteristic** | **Predator** | | | | | **Prey** | | | | |
| --- | --- | --- | --- | --- | --- | --- | --- | --- | --- | --- |
|  | Exp1 | Exp2 | Exp3 | Exp4 | Exp5 | Exp1 | Exp2 | Exp3 | Exp4 | Exp5 |
| Average speed (cells / time step) | 4 | 4.2 | 4.1 | ... | ... | 3.1 | 3.2 | 3.1 | ... | ... |
| Average level of energy (units) | 445 | 448 | 432 | n/a | n/a | 278 | 271 | 268 | n/a | n/a |
| Maximum level of energy (units) | 1000 | 1000 | 1000 | n/a | n/a | 650 | 650 | 650 | n/a | n/a |
| Average number of reproduction action during life | 1.14 | 1.21 | 1.18 | ... | ... | 1.49 | 1.37 | 1.41 | ... | ... |
| Average length of life (time steps) | 9 | 10 | 10 | 4 | 5 | 13 | 13 | 12 | 4 | 5 |
| Number of individuals  (in thousands) | 31  ($\pm$7) | 36  ($\pm$9) | 33  ($\pm$11) | 38  ($\pm$4) | 37  ($\pm$5) | 307  ($\pm$24) | 293  ($\pm$28) | 285  ($\pm$17) | 261  ($\pm$8) | 266  ($\pm$9) |
| Level of patchiness (STC) | 0.26$\pm$  (0.05) | 0.25$\pm$  (0.04) | 0.84$\pm$  (0.11) | 0.92$\pm$  (0.08) | 0.38$\pm$  (0.08) | 0.24$\pm$  (0.07) | 0.26$\pm$  (0.06) | 0.82$\pm$  (0.12) | 0.9$\pm$  (0.05) | 0.41$\pm$ (0.09) |

**2-3- Measure for cluster quality**

In order to explore the causality of species formation, we investigated the conditions that lead to the emergence of strong phenotypic/genotypic clusters. We investigated whether our species concept implemented in EcoSim is consistent with the genotypic cluster definition. To achieve this we analyzed the degree of compactness and isolation of the generated clusters of genomes, called species-clusters. Then, we compared the species-clusters obtained at selected time steps (12,000, 14,000, 16,000, 18,000 and 20,000) with clusters obtained by K-means-clustering and random-clustering. The speciation mechanism implemented in EcoSim (see above) can be viewed as an on-line hierarchical clustering process, with each species being a cluster of genomes. Since clustering is a difficult and time-consuming task, and therefore no exact (i.e. with guaranty of optimality) algorithm exists, it is impossible to apply it to the whole population of individuals at each time step. For example, at some time steps, EcoSim supports more than 500,000 individuals. We have therefore chosen a heuristic hierarchical approach in which clustering is done greedily along the evolutionary process. Therefore, at a given time step, only a small subset of individuals is effectively clustered by our species-splitting mechanism (the ones that are members of species that split at this time step). For ease of comparison, the k-means-clusters were obtained by directly applying a k-means clustering algorithm [34], with k being the number of clusters at that particular time step, to the whole population of genomes using the same number of clusters. As a reference for a lower bound of cluster quality, we also conducted random clustering using the same number of clusters k and randomly assigning each individual to one of the clusters.


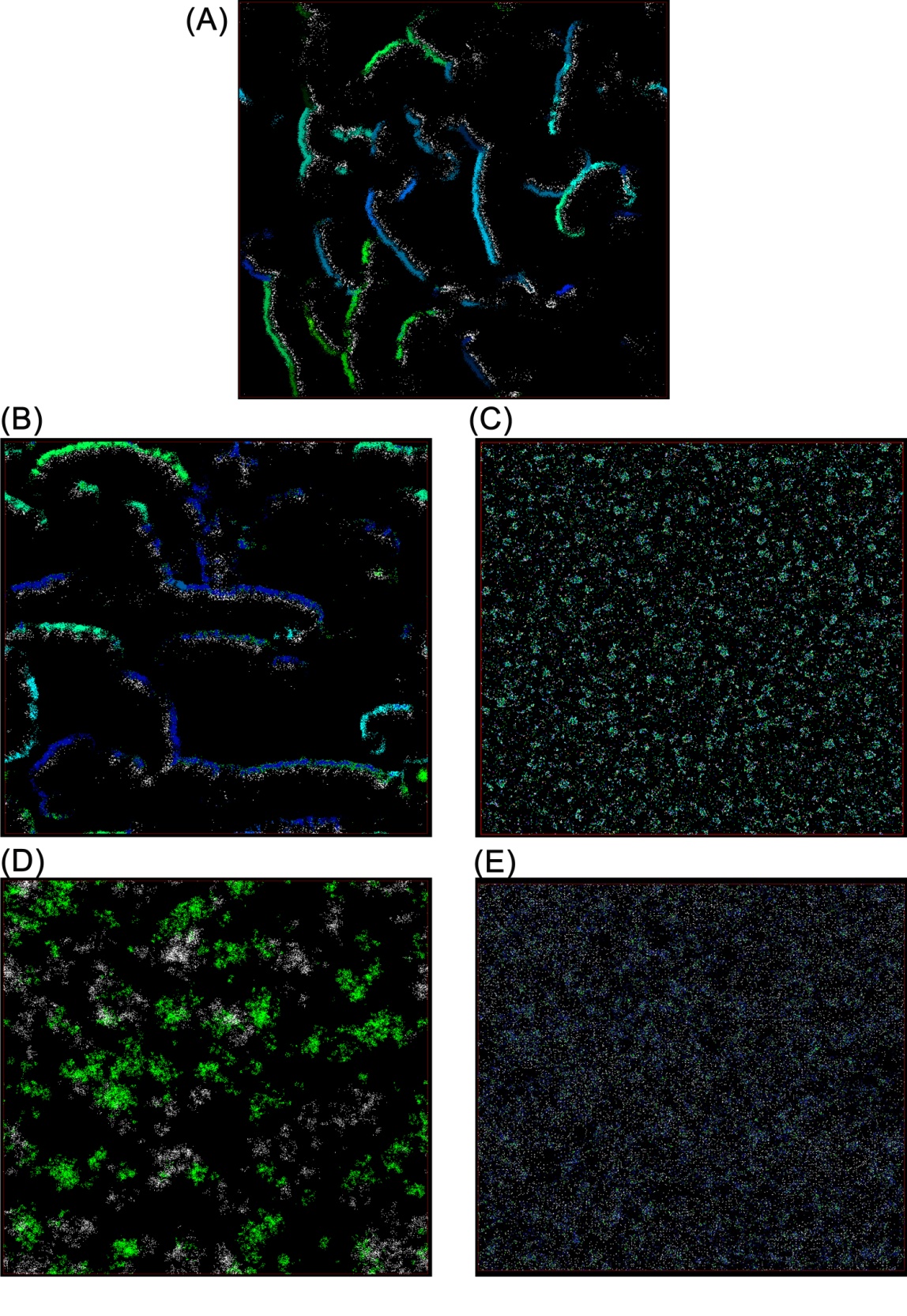


**Figure A.** **Spatial distribution of individuals in the different versions of simulation.** (**A**) Selection, Enforced Reproductive Isolation, and Low Dispersal experiment (**B**) Selection and Low Dispersal experiment (**C**) Selection and High Dispersal experiment (**D**) No Selection and Low Dispersal experiment (**E**) No Selection and High Dispersal experiment. Different colors stand for different prey species. Predators are represented in white.

To evaluate the separation between clusters and their compactness we calculated the distance between each species’ genetic center, which represents the average of all individual genomes, and the individual the farthest from the center in term of genomic similarity. Furthermore, we calculated the mean and standard deviation of the distances between the genetic centers of each pair of species at four different time steps (10,000, 12,000, 14,000, 16,000). In addition, we used the Davies-Bouldin index [35], a commonly used method for measuring the quality of clustering algorithms both in term of separation between clusters and their compactness. We also used this index to compare the quality of different clustering results for the five experiments.

To analyze the level of reproductive isolation between species obtained in the five experiments, we measured the percentage of mating events that generate hybrid offspring (that is, offspring for which each parent is a member of a different species) across all mating events. These analyses were performed on all replicates of the five experiments separately for the first 10,000 time steps and for the time steps between 10,000 and 20,000. This approach was used because the behavior of the system takes time to stabilize.

**3- Results**

**3-1- Conditions leading to genotypic clusters**

In all the experiments that involved strong natural selection, the distance between genetic clusters was high for the species-clusters and k-means-clusters but significantly smaller for random-clusters (see Table E, one-way ANOVA, *P* < 0.001: Tukey post hoc test, *P* < 0.05). Moreover, the species-clusters were always more separated than the k-means-clusters, although this was not significant (one-way ANOVA, *P* > 0.05). When measuring the distance of the farthest individual from the genetic center of species (see Table E) we found that in all experiments with natural selection, the k-means-clusters and the species-clusters are much more compact than the random-clusters, with the difference being statistically significant (one-way ANOVA, *P* < 0.0001: Tukey post hoc test, *P* < 0.05). Furthermore, we also observed that the compactness of the species-clusters is always greater than that of the k-means-clusters, even though the difference is not significant (one-way ANOVA, *P* > 0.05).

In the **No Selection and High Dispersal** experiment, the separation between k-means-clusters and species-clusters is still higher than that of random-clusters (Table E), albeit the difference is not significant (one-way ANOVA, *P* > 0.05). We can also observe that the compactness is much less stringent than in the experiments with natural selection and does not vary significantly between the three cluster types (one-way ANOVA, *P* > 0.05). In the **No Selection and Low Dispersal** experiment, when natural selection has been deactivated but there is still geographic isolation due to the strong grouping of related individuals, the k-means and species clusters are statistically more compact and specially separated than random ones, but the difference is less prominent than when natural selection is involved. The distance between clusters is smallest for the random-clusters, although this is not statistically significant (one-way ANOVA, *P* > 0.05). The difference with respect to distance between clusters in the k-means-clusters and species-clusters is also not significant (one-way ANOVA, *P* > 0.05). However, the k-means-clusters and the species-clusters are much more compact than the random-clusters, and the difference is significant (one-way ANOVA, *P* < 0.001: Tukey post hoc test, *P* < 0.05).

Furthermore, in the experiments with natural selection, the Davies-Bouldin index indicates that the species clusters are in general more compact and isolated than k-means-clusters, although it is not statistically significant (one-way ANOVA, *P* > 0.05). The Davies-Bouldin index for random-clusters is significantly lower than that of the species-clusters and k-means-clusters (one-way ANOVA, *P* < 0.0001: Tukey post hoc test, *P* < 0.05). These findings show that virtual species in the experiments with natural selection reflect concepts and behaviors observed in natural populations. The species that emerge successively in the experiments with natural selection are at least as well separated and at least as compact as those obtained by a direct clustering of the genomes. This suggests that the heuristic clustering method we used to identify species closely-follows the logic of the genotypic cluster definition.

In the **Selection and High Dispersal** experiment, where any two individuals can mate (since there is no extrinsic restriction or geographic barrier to reproduction) and the individuals are not grouped by parental relationships (Figure A(C)), a distinct separation between species was nevertheless observed. This is a confirmation of the strong impact of natural selection on the formation of distinct clusters. The Davies-Bouldin index also confirms that the performances of the three cluster types are almost the same in the case of the **No Selection and High Dispersal** experiment, with the difference between them not being significant (one-way ANOVA, *P* > 0.05). This means that, in the absence of natural selection and geographic isolation, the quality of species-clusters and k-mean-clusters is only marginally better than that of random clusters, showing that the effect of genetic drift alone is not sufficient to create well separated and compact groups of genomes.

In the **No Selection and Low Dispersal** experiment, the quality of the random-clusters did not significantly differ from k-means-clusters (two-sample, two-tailed independent Student’s *t*-test, p-value = 0.06). However, species-clusters perform significantly better than random clusters (two-sample, two-tailed independent Student’s *t*-test, p-value = 0.04). The absence of natural selection seems, therefore, to be partially compensated by the addition of geographic isolation to the ecosystem, which maintains a low level of gene flow between populations and allows divergence between groups of organisms. However, when comparing these results with those of the **Selection and High Dispersal** experiment, it clearly appears that the effect of natural selection is much more important than the effect of reduced gene flow (one-way ANOVA, *P* < 0.001: Tukey post hoc test, *P* < 0.05).

**Table E. Exploring the causality of species formation.** Average and standard deviation of the distance of the farthest individual from its cluster genetic center’s (cluster-Individual), the distance between genetic centers of two clusters (cluster-cluster), and the Davies-Bouldin index in different versions of simulation. For the cluster-Individual distance and the Davies-Bouldin index, the lower the value the better the cluster is.

|  | cluster-Individual | | cluster-cluster | | Davies-Bouldin Index | |
| --- | --- | --- | --- | --- | --- | --- |
|  | Mean | std | Mean | std | Mean | std |
| a) Selection, Enforced Reproductive Isolation, and Low Dispersal |  |  |  |  |  |  |
| k-means-clusters | 23.2 | 9.7 | 64.9 | 6.7 | 2.1 | 0.4 |
| Species-clusters | 23.4 | 8.3 | 66.1 | 10 | 2.1 | 0.4 |
| Random-clusters | 68.6 | 3.9 | 17 | 4.5 | 7.8 | 0.7 |
| b) Selection and Low Dispersal |  |  |  |  |  |  |
| k-means- cluster | 25.1 | 9.8 | 63 | 11.9 | 2 | 0.4 |
| Species-clusters | 23.6 | 9.7 | 64.5 | 9.8 | 1.9 | 0.5 |
| Random- cluster | 62.6 | 2.8 | 17.2 | 4 | 8 | 0.4 |
| c) Selection and High Dispersal |  |  |  |  |  |  |
| k-means- cluster | 28.6 | 10.3 | 56.3 | 9 | 2.2 | 0.5 |
| Species-clusters | 26.2 | 9.2 | 57.4 | 9.2 | 2.1 | 0.6 |
| Random- cluster | 65.4 | 2.5 | 16.9 | 3 | 7.8 | 0.3 |
| d) No Selection and High Dispersal |  |  |  |  |  |  |
| k-means- cluster | 70.9 | 31.6 | 44.3 | 17.4 | 4.2 | 0.5 |
| Species-clusters | 70.4 | 30 | 43.9 | 16.5 | 4.1 | 0.5 |
| Random- cluster | 87.7 | 8.7 | 32.5 | 9.8 | 4.5 | 0.4 |
| e) No Selection and Low Dispersal |  |  |  |  |  |  |
| k-means- cluster | 58.2 | 20.8 | 49.4 | 19.9 | 3.5 | 0.6 |
| Species-clusters | 60.6 | 22.7 | 50.4 | 21.2 | 3.4 | 0.7 |
| Random- cluster | 93.9 | 10.7 | 36.2 | 9.6 | 4 | 0.5 |

**3-2 Rate of Hybridization**

For the first 10,000 time steps, the percentage of mating leading to hybrids was 17% (std 5%) for **Selection, Enforced Reproductive Isolation, and Low Dispersal**, 87% (std 6%) for **Selection and Low Dispersal**, 89% (std 7%) for **Selection and High Dispersal**, 91% (std 3%) for **No Selection and Low Dispersal** and 92% (std 3%) for **No Selection and High Dispersal**. The only experiment statistically different from the others (one-way ANOVA, *P* < 0.001: Tukey post hoc test, *P* < 0.05) is **Selection,** **Enforced Reproductive Isolation, and Low Dispersal**. However, for the next 10,000 time steps, after the stabilization of the simulations that involve the behavioral model, the percentage of hybrid progeny is 20% (std 7%) for **Enforced Reproductive Isolation, and Low Dispersal**, 25% (std 5%) for **Selection and Low Dispersal**, 26% (std 4%) for **Selection and High Dispersal**, 94% (std 2%) for **No Selection and Low Dispersal** and 95 (3%) for **No Selection and High Dispersal**. The experiments with natural selection are not significantly different from each other, but they are all different from the two experiments without natural selection (one-way ANOVA, *P* < 0.0001: Tukey post hoc test, *P* < 0.05).

**References**

1. Holland JH (1992) Adaptation in Natural and Artificial Systems: An Introductory Analysis with Applications to Biology, Control and Artificial Intelligence . Cambridge, MA: MIT Press.
2. Mitchell M (1996) An Introduction to Genetic Algorithms. Cambridge, MA: MIT Press.
3. Kallel L, Naudts B, Rogers A (2001) Eds. Theoretical Aspects of Evolutionary Computing. Berlin: Springer.
4. Packard NH (1989) Intrinsic adaptation in a simple model for evolution. Artif Life 141.
5. Channon AD, Damper RI (1998) Perpetuating evolutionary emergence, in From Animals to Animats 5: Proceedings of the Fifth International Conference on Simulation of Adaptive Behavior (SAB98), Zurich, R. Pfeifer, B. Blumberg, J.-A. Meyer and S. Wilson (eds.), MIT Press: Cambridge, MA, 534–539.
6. Goldberg DE, Richardson J (1987) Genetic algorithms with sharing for multimodal function optimization, Proceedings of the Second International Conference on Genetic Algorithms on Genetic algorithms and their application, Cambridge, Massachusetts, 41-49.
7. Mahfoud S (1993) Simple analytical models of genetic algorithms for multi-modal function optimization, Technical Report, Department of Computer Science, University of Illinois at Urbana-Champaign, Urbana, IL, USA, Illinois Genetic Algorithm Laboratory Report No. 94005.
8. Grefenstette JJ (1999) Evolvability in dynamic fitness landscapes: a genetic algorithm approach. *Evolutionary Computation, 1999. CEC 99.* Proceedings of the 1999 Congress on 3, 2031–2038.
9. Chen Y (2006) Extending the Scalability of Linkage Learning Genetic Algorithms: Theory & Practice*.* Berlin: Springer.
10. Pelikan M, Goldberg DE, Lobo FG (2002) A Survey of Optimization by Building and Using Probabilistic Models. Comput Optim Appl 21: 5–20.
11. Pelikan, M (2005) Hierarchical Bayesian Optimization Algorithm: Toward a New Generation of Evolutionary Algorithms. Berlin: Springer.
12. Channon AD, Damper RI (2000) Towards the evolutionary emergence of increasingly complex advantageous behaviours. Int J Syst Sci 31: 843–860.
13. Bedau MA, Packard N (1992) Measurement of evolutionary activity, teleology, and life Langton, C., Taylor, C., Farmer, D. & Rasmussen, S. (Eds.), Artificial life II, Addison–Wesley, Reading.
14. Yaeger L (1992) Computational genetics, physiology, metabolism, neural systems, learning, vision, and behavior or PolyWorld: life in a new context. In Proc. Artificial Life III, Santa Fe Institute Studies in the Sciences of Complexity, vol. 17, Redwood City, CA: Addison-Wesley, 263–298.
15. S. Gavrilets, *Fitness landscapes and the origin of species (MPB-41)* (Princeton University Press, 2004).
16. Gavrilets S, Vose A,Barluenga M, Salzburger W, Meyer A (2004) Case studies and mathematical models of ecological speciation. 1. Cichlids in a crater lake. Molecular Ecology 16: 2893–2909.
17. Dieckmann U, Doebeli M (1999) On the origin of species by sympatric speciation. *Nature* 400(6742): 354–357.
18. Kirkpatrick M, Nuismer SL (2004) Sexual selection can constrain sympatric speciation. Proc Biol Sci 271: 687–693.
19. Bolnick DI (2006) Multi-species outcomes in a common model of sympatric speciation. J Theor Biol 241: 734–744.
20. Drossel B, Mckane A (2000) Competitive speciation in quantitative genetic models. J Theor Biol 204: 467–478.
21. Doebeli M, Blok HJ, Leimar O, Dieckmann U (2007) Multimodal pattern formation in phenotype distributions of sexual populations. Proc Biol Sci 274: 347–357.
22. Doebeli M, Dieckmann U (2003) Speciation along environmental gradients, Nature 421: 259–264.
23. Higashi M, Takimoto G, Yamamura N (1999) Sympatric speciation by sexual selection. Nature 402: 523–526.
24. Takimoto G, Higashi M, Yamamura N (2000) A deterministic genetic model for sympatric speciation by sexual selection. Evolution 54: 1870–1881.
25. Gavrilets S,Vose A (2005) Dynamic patterns of adaptive radiation. PNAS 102: 18040 18045
26. Gavrilets S, Losos JB (2009) Adaptive radiation: contrasting theory with data. Science

323: 732–737.

1. Thibert-Plante X, Hendry AP (2011) Factors influencing progress toward sympatric speciation. J Evol Biol 24: 2186–2196.
2. Thibert-Plante X, & Hendry AP (2011) The consequences of phenotypic plasticity for ecological speciation. J Evol Biol 24: 326–342.
3. Débarre F (2012) Refining the conditions for sympatric ecological speciation. J Evol Biol 25: 2651–2660.
4. Allen B, Nowak MA, Dieckmann U (2013) Adaptive dynamics with interaction structure. Am Nat 181:E 139–E163.
5. Metz JAJ (2008) Fitness. Encyclopedia of ecology 2: 1599-1612.
6. Metz JAJ, Geritz SAH, Meszena G, Jacobs FJA, Van Heerwaarden JS, others (1996) Adaptive dynamics, a geometrical study of the consequences of nearly faithful reproduction. Stochastic and spatial structures of dynamical systems 45: 183–231
7. Parrott L, Proulx R, Thibert-Plante X (2008) Three-dimensional metrics for the analysis of spatiotemporal data in ecology. Ecol Inform 3: 343–353.
8. MacKay DJC (2003) Information Theory, Inference and Learning Algorithms. New York: Cambridge University Press.
9. Davies DL, Bouldin DWA (1979) Cluster Separation Measure. IEEE Trans Pattern Anal Mach Intell PAMI-1: 224–227.
